# How does contemporary selection shape oak phenotypes?

**DOI:** 10.1111/eva.13082

**Published:** 2020-08-14

**Authors:** Hermine Alexandre, Laura Truffaut, Etienne Klein, Alexis Ducousso, Emilie Chancerel, Isabelle Lesur, Benjamin Dencausse, Jean‐Marc Louvet, Gérard Nepveu, José M. Torres‐Ruiz, Frédéric Lagane, Brigitte Musch, Sylvain Delzon, Antoine Kremer

**Affiliations:** ^1^ INRAE University of Bordeaux BIOGECO Cestas France; ^2^ INRAE BIOSP, Site Agroparc Avignon France; ^3^ Helix Venture Merignac France; ^4^ INRAE University of Nancy SILVA Champenoux France; ^5^ INRAE University of Clermont‐Auvergne PIAF Clermont‐Ferrand France; ^6^ ONF INRAE BIOFORA Ardon France; ^7^ INRAE University of Bordeaux BIOGECO Pessac France

**Keywords:** fitness, *Quercus petraea*, *Quercus robur*, second theorem of selection, selection gradients

## Abstract

Most existing forests are subjected to natural and human‐mediated selection pressures, which have increased due to climate change and the increasing needs of human societies for wood, fibre and fuel resources. It remains largely unknown how these pressures trigger evolutionary changes. We address this issue here for temperate European oaks (*Quercus petraea* and *Q. robur*), which grow in mixed stands, under even‐aged management regimes. We screened numerous functional traits for univariate selection gradients and for expected and observed genetic changes over two successive generations. In both species, growth, leaf morphology and physiology, and defence‐related traits displayed significant selection gradients and predicted shifts, whereas phenology, water metabolism, structure and resilience‐related traits did not. However, the direction of the selection response and the potential for adaptive evolution differed between the two species. *Quercus petraea* had a much larger phenotypic and genetic variance of fitness than *Q. robur*. This difference raises concerns about the adaptive response of *Q. robur* to contemporary selection pressures. Our investigations suggest that *Q. robur* will probably decline steadily, particularly in mixed stands with *Q. petraea*, consistent with the contrasting demographic dynamics of the two species.

## 1 INTRODUCTION

Most forests worldwide are renewed by natural processes, the exception being artificially planted woodlands, which account for 7% of the world's forests (FAO, 2015). The remaining primary and secondary forests are regenerated by means of seedlings resulting from open pollination between mature trees. Natural processes dominated by competition for light, water and nutrients, and environmental disturbances, exert a strong influence during the early stages of development of the renewed forest stand. Human activities, in the form of silvicultural regimes of various intensities, depending on tree species and country, subsequently superimpose over natural selection regimes, except in primary forests. Thus, most existing forests are subjected to both natural and human‐mediated selection pressures, which have increased in intensity due to climate change and the increasing needs of human societies for wood, fibre and fuel resources. However, little is known about how these pressures genetically shape tree phenotypes. We addressed this question for temperate European oaks (*Quercus petraea* and *Q. robur*), which are grown at a continent‐wide scale and are subjected to a combination of natural and human selection pressures. We investigated whether contemporary selection pressures may contribute to a marked and heritable shift in the phenotypic traits of oak trees. Large‐scale surveys in European forests emphasized clear phenotypic trends for some traits that have been related to climatic drivers. Continuous increases of growth have been recorded during the last century but also at contemporary time scales (Becker, Nieminen, & Geremia, 1994; Maes et al., 2019; Spiecker, 2003). The timing of bud burst has steadily been shifted to earlier dates as a result of temperature increases (Menzel et al., 2006; Vitasse, Delzon, Dufrene, et al., 2009). During the last two decades, the yearly seed crop increased as well in temperate oaks (Caignard et al., 2017). It is, however, unknown whether these phenotypic trends are driven by genetic shifts or only by plasticity responses. It is also unknown whether they contribute to higher or lower fitness. Answers to these questions will be provided by the comparison of the direction and rate between genetic and phenotypic changes occurring at contemporary time scales.

Genetic shifts in forest trees are generally predicted under artificial conditions, such as common garden experiments, designed to encompass the differences between populations that have amassed over the multiple generations since their divergence. In this study, we aimed to monitor these predicted changes in the “real world,” in situ, in the conditions in which the oak forests of today are experiencing selection pressures, and over short time scales (e.g. over two successive generations).

Interest in methods for predicting genetic changes in situ has recently increased, and there has been progressed in the development of statistical and genomic toolkits (Gienapp et al., 2017; Pujol et al., 2018), despite the discrepancies between predicted and observed evolutionary shifts occasionally mentioned (Charmantier, Garrant, & Kruuk, 2014; Kruuk, Slate, & Wilson, 2008). These approaches are based on methods (single and multivariate breeder's equation, Lande, 1979; Walsh & Lynch, 2018 p. 479) used in artificial selection schemes in which directional culling selection drives genetic changes over successive breeding populations (Falconer, 1995; Lynch & Walsh, 1998). Their use in ecological settings in which natural selection predominates has been improved by the availability of new statistical methods (Kruuk, 2004; Stinchcombe, Simonsen, & Blows, 2014; de Villemereuil, 2018) and provides opportunities to explore possible genetic changes in response to the environmental changes currently underway. Transmissible evolutionary changes in phenotypic traits due to selection can be predicted if the following criteria are met (Robertson, 1966; Walsh & Lynch, 2018 p. 146): (a) the trait is correlated with fitness and (b) this correlation comprises a genetic (additive) component linking the trait to fitness. If these conditions are met, then natural or human selective pressures operating at a given generation can contribute to changes that are transmitted to the next generation, eventually leading to evolutionary shifts. The first of these two conditions is usually addressed by assessing selection gradients or selection differential in natural populations (Hendry, 2016; Kingsolver, Diamond, Siepielski, & Carlson, 2012), whereas the second is addressed with the secondary theorem of selection (Morrissey et al., 2012; Price, 1970), which provides an estimate of the expected response to selection. Implicitly, both conditions require the traits of interest and fitness to display genetic variation in the population studied. Indeed, a key feature of selection is that it requires evolutionary potential and genetic variation for fitness within the population (Bonnet, Morrissey, & Kruuk, 2019; Hendry, Schoen, Wolak, & Reid, 2018). We implemented these approaches in the context of oak forests, addressing some of the biological and ecological constraints identified concerning their use in plants or trees (Bontemps, Lefevre, Davi, & Oddou‐Muratorio, 2016; Castellanos, Gonzalez‐Martinez, & Pausas, 2015; Sedlacek et al., 2019). Constraints raise from the peculiar demographic, spatial and genetic structures of forest stands conducted under even‐aged regimes. Oak populations exhibit usually spatial and genetic structures (Streiff, Ducousso, & Kremer, 1998) which can lead to nonindependence of genetic and environmental effects, and to competition effects, and further bias the estimation of the required genetic parameters. Furthermore, assessing fitness in oak stands raises sampling challenges within the recruited cohort of oak seedlings that we have addressed by estimating individual fecundities after accounting for the spatial distribution of the parents and the offspring (MEMM framework, Oddou‐Muratorio, Gauzere, Bontemps, Rey, & Klein, 2018) instead of simply recording the realized reproductive success in the sample offspring. Within this theoretical framework, we tried to identify the traits likely to be exposed to genetic change due to ongoing natural and human selection pressures. We dissected oak phenotypes into numerous functional and ecologically relevant traits relating to growth, phenology, water metabolism, morphology, secondary metabolism, resilience, defence and wood structure. Some of these traits are repeatedly advocated to contribute to fitness and adaptation of trees (Anderegg, 2015; Funk et al., 2017; McKown et al., 2014), but their actual correlation with fitness has been assessed only rarely *in natura* (Duputie, Rutschmann, Ronce, & Chuine, 2015). We proceeded in three steps. We first explored whether there was a potential for genetic changes to occur, by assessing fitness and its genetic variation. We then tried to identify traits correlated with fitness, by estimating selection gradients and differentials. Finally, we checked whether there was genetic support for this correlation, ensuring that selection would contribute to genetic changes in the next generation. The main purpose of this study was to evaluate qualitative rather than quantitative genetic changes, by targeting the direction of changes, rather than the rate of change. We applied our three‐step approach to a mixed oak forest comprising two white oak species (*Quercus petraea* and *Q. robur*) with different demographic dynamics over two generations (Truffaut et al., 2017). We investigated whether these dynamics were related to different responses to the ongoing natural and human‐driven selection.

## 2 MATERIALS AND METHODS

### 2.1 Study population

The study population was a mixed forest stand composed of *Q. petraea* and *Q. robur* covering 5.19 ha in the Petite Charnie State Forest (latitude: 48.086°N; longitude: 0.168°W) in France, which has been intensively studied over the last 30 years (Truffaut et al., 2017). Phenotypic traits were monitored over two successive generations. The first generation (G1) comprised 422 (196 *Q. petraea* and 226 *Q. robur*) trees that were about 100 years old at the time they were cut, between 1989 and 2001. Before the final clear cut in 2001, the stand was progressively opened up by removal cuts practiced in 1992, 1993 leaving at the end 116 *Q. petraea* and 143 *Q. robur* trees. The final clear cut took place over a period of 3 years (1999, 2000 and 2001), to facilitate the harvesting and manipulation of log samples for later assessments of the wood and tree anatomy. Before they were cut, the trees mated between 1989 and 2001, giving rise to a second generation (G2), as illustrated in Supporting Information S1. From 1995 onwards, G1 trees were grafted in a conservation collection located in Guéméné‐Penfao in North‐West France (latitude: 47.631°N; longitude: 1.892°W), before being finally cut. The density of G2 saplings was extremely high, and a systematic sampling of 2,510 saplings (one sapling every 3–6 m) was implemented in G2 for the reconstruction of parentage in 2014 (Truffaut et al., 2017). All the G1 trees and the sampled G2 saplings were mapped by recording their GPS coordinates, with postprocessed differential correction (G1 trees mapped in 1992 and G2 trees in 2014). Indicator values for key ecological variables were collected in a floristic survey conducted in 1992: pH, soil moisture, carbon‐to‐nitrogen ratio and organic matter content. These variables were downscaled to the spatial resolution of each G1 and G2 tree (Truffaut et al., 2017). Phenotypic assessments of G2 trees were also conducted, from 2014 onwards (Supporting Information S2). The recruitment period of G2, up to the phenotypic monitoring stage, thus extended from 1989 to 2014.

### 2.2 Assessment of phenotypic traits

Phenotypic traits were monitored in G1 and G2 as follows (Supporting information S2):

1. In G1, between 1989 and 2001, when the trees were between 90 and 100 years old, 56 traits correlated with major functional and ecophysiological classes (growth, reproduction, phenology, physiology, resilience, wood structure, leaf morphology and plant defence) were measured (Table 1). Assessments were made on all G1 trees. Detailed descriptions of trait assessments are provided in Supporting information S2 and are also available from (Alexandre et al., 2020), which used the same nomenclature.
2. In G2, between 2014 and 2017, when the trees were between 14 and 28 years old, 11 traits were assessed on a sample of 370 *Q. petraea* and 390 *Q. robur* saplings (Supporting information S3). These traits corresponded to the following functional classes:
  - Growth: total height (HGHT) and circumference (CIRC).
  - Phenology: time at leaf unfolding (LU).
  - Physiology and water metabolism: specific leaf area (SLA), mean leaf area (MLA), carbon content of leaves (C ), nitrogen content (N), carbon/nitrogen ratio (C/N), carbon isotopic composition (δ^13^C) and nitrogen isotopic composition (δ^15^N).
  - Wood structure: wood density (WD) recorded on increment cores with an X‐ray image calibration procedure.

**TABLE 1:** Description of the traits

Trait class	Trait acronym	Trait units	Trait definition	G1	G2
Growth	CIRC	cm	Circumference of the stem at breast height	*	*
	HGHT	cm	Total height of the tree	*	*
	RSURF	mm^2^	Mean yearly ring surface	*	
	RWDTH	mm	Mean yearly ring width	*	
Phenology	LU	nb of days/score	Julian day of leaf unfolding	*a	*b
	LS	nb of days	Julian day of leaf senescence	*	
	GSL	nb of days	Length of growing season	*	
	FFLW	nb of days	Julian day of female flowering	*	
	MFLW	nb of days	Julian day of male flowering	*	
	MAR	score	Marcescence	*	
Physiology	C	g/kg	Carbon content in leaves	*c	*
	C/N	ratio	Carbon/Nitrogen ratio	*c	*
	δ^13^C	‰	Leaf carbon isotope (^13^C) composition	*c	*
	δ^15^N	‰	Leaf nitrogen isotope (^15^N) composition	*c	*
	MLA	cm^2^	Mean leaf area	*c	*
	N	g/kg	Nitrogen content of leaves	*c	*
	SLA	m^2^/kg	Specific leaf area	*c	*
Resilience	REC	ratio	Recovery (increased ring growth relative to the growth during a stress episode)	*	
	REL	ratio	Resilience (ability of the tree to reach prestress episode ring growth)	*	
	RET	ratio	Resistance (inverse of ring growth reduction during a stress episode)	*	
Structure	WD	kg/m^3^	Wood density	*d	*e
Leaf morphology	BS	score	Basal lamina shape	*	
	HR	score	Pubescence	*	
	LDR	%	Lobe depth ratio	*	
	LL	mm	Lamina length	*	
	LW	mm	Lobe width	*	
	LWR	%	Lobe width ratio	*	
	NL	count	Number of lobes	*	
	NV	count	Number of intercalary veins	*	
	OB	%	Obversity	*	
	PL	mm	Petiole length	*	
	PR	%	Petiole length ratio	*	
	PV	%	Percentage of venation	*	
	SW	mm	Sinus width	*	
	WP	mm	Lamina length (largest width)	*	
Defence	CNFL	µg/g	Coniferaldehyde (VC)	*	
	CSTG	%	Castalagin (E)	*	
	CSTL	%	Castaline (E)	*	
	CWSK	µg/g	C‐whisky lactone (VC)	*	
	EGNL	µg/g	Eugenol (VC)	*	
	ELAC	µg/g	Ellagic acid (E)	*	
	ELTOT	µg/g	Total ellagitannin (E)	*	
	GRDN	%	Grandinine (E)	*	
	MVL	µg/g	Mevalonic lactone (VC)	*	
	PNTL	µg/g	Pantolactone (VC)	*	
	ROBA	%	Roburine A (E)	*	
	ROBB	%	Roburine B (E)	*	
	ROBC	%	Roburine C (E)	*	
	ROBD	%	Roburine D (E)	*	
	ROBE	%	Roburine E (E)	*	
	SYRG	µg/g	Syringaldehyde (VC)	*	
	TWSK	µg/g	T‐whisky lactone (VC)	*	
	VNL	µg/g	Vanillin (VC)	*	
	VSCG	%	Vescalagin (E)	*	
	VSCL	%	Vescaline (E)	*	
	X2PHL	µg/g	2‐phenylethanol (VC)	*	

Abbreviations: a, assessed as the number of days; b, assessed by a scoring system; c, assessed in the grafted conservation collection; d, assessed as infradensity; e, assessed by X‐ray; E, ellagitannin; VC, volatile compound.

These traits were also assessed in G1 trees, but at different ages. Table 1 summarizes all the traits assessed in G1 and G2. The physiological traits assessed on G1 trees were recorded on the grafted clonal copies in the conservation orchard rather than *in natura* on the standing trees (Table 1).

### 2.3 Genotyping

All G1 trees still present in 1998 (260 trees), and all G2 saplings (2,510) were genotyped in 2015 with a set of 82 SNPs to estimate reproductive success (see the next paragraph), and for species assignment (Truffaut et al., 2017, Methods S3 and Table S1). SNP genotyping was performed with the MassARRAY^®^ System (Agena Bioscience™) and iPLEX^®^ chemistry. SNP arrays were used in a previous study (Truffaut et al., 2017) for parentage analysis, and the same data set was used here for the estimation of reproductive success (section 2.5).

All G1 trees were also genotyped with a set of 15,000 SNP markers (15,274 in *Q. petraea* and to 16,408 in *Q. robur*) derived from targeted sequence capture (Lesur et al., 2018). This data set was used for the assessment of genomic relatedness between G1 trees required for estimation of the genetic variances/covariances of the phenotypic traits and fitness (section 2.6, and [Alexandre et al., 2020]). We previously showed (Lesur et al., 2018) that the genomic relatedness estimated with these 15,000 SNPs was consistent with pedigree relationships in a validation sample of trees of known pedigree.

### 2.4 Fitness assessment

The peculiar ecological setting of forest stands undergoing even‐aged silvicultural regimes raises technical and experimental concerns regarding the estimation of fitness and genetic parameters that are targeted in this study. Most of these concerns are related to the spatial distribution of trees in the two generations. G1 and G2 trees are not distributed uniformly, and local densities are heterogeneous (Figure S2–S4 in Supporting information S3). Such distributions may impact the estimation of reproductive success due to the sampling strategy of G2 saplings used for parentage analysis and due to border effects. Furthermore, variation of spacing between trees may result in variation of competition effects which introduce noise for the estimation of selection gradients or genetic variances/covariances of different traits. Earlier genetic studies conducted in this stand underpinned also the existence of spatial genetic structures due to repeated cycles of natural regeneration (Bacilieri, Labbe, & Kremer, 1994; Streiff et al., 1998), thus generating nonindependence between genetic and environmental effects. In the next sections, we attempted to account explicitly for these sources of variation in the methods and models used for estimating fitness, selection gradients and genetic variances/covariances.

The forest stand at La Petite Charnie studied here is managed under a traditional even‐aged silvicultural regime (Supporting Information S1). Under this system, generations do not overlap, and all the trees of the stand are of about the same age and grow together until the final cut of the stand, which may occur after 80–250 years in oak stands, depending on their location (Jarret, 2004). About 10 years before the final cut, a seed cut takes place, to enhance reproduction through natural crossing. The seed cut opens up the canopy, providing open‐pollinated seeds of the next generation with sufficient light to ensure germination and growth. The next generation is thus assembled from the seedlings emerging in the 10 successive years following the seed cut. Seedling densities at the renewal stage may exceed 100,000/ha, but are strongly decreased by natural selection and competition, to reach values of 2,000–4,000 trees/ha at the age of 20 years (Supporting information S1). Thinning operations by humans subsequently decrease densities further, to about 100/ha by the time of the next final seed cut. Under this mixed natural and human‐mediated selection regime, the fitness W of a tree can be assessed by determining its effective reproductive success (RS) as the total number of offspring still alive at the time of reproduction in the next generation.

We measured RS of G1 trees by assessing the number of living G2 offspring they produced and still living at the age of 14–28 years, as male or female parent. At this stage, more than 95% of the regenerating trees have already been eliminated (Supporting information S1), so RS provides a very close proxy for realized fitness, taking into account both the fecundity of the parent tree and the survival of its offspring. However, the genotyped G2 saplings were a sample of this cohort, collected according to a predetermined spatial sampling design. We therefore performed a statistical analysis to eliminate the effects of the spatial geometry of the experimental design. The relative positions of the seedling sampling sites, the locations of the adult trees and of the plot borders affect the observed reproductive success obtained directly from a CERVUS‐like parentage analysis (Oddou‐Muratorio et al., 2018). As described by Oddou‐Muratorio et al. (2018) and Tonnabel et al. (submitted), we used the MEMM_seedlings framework to infer, for each adult, an “effective fecundity” referred to as “effective reproductive success” below, for the sake of simplicity. This effective fecundity was inferred from the observation of seedling genotypes, considering the locations of the seedlings and the genotypes and locations of the adults to be known (Supporting information S4.1). RS calculated in this manner is a relative measure of effective reproductive success (i.e. mean value of 1 for all G1 trees) and was calculated for each tree by averaging the RS values of its female and its male parents with equal weightings. Robledo‐Arnuncio and Garcia (2007) and Klein, Bontemps, and Oddou‐Muratorio (2013) showed that the spatially explicit mating model (such as MEMM_seedling) was robust to irregular sampling designs and to spatially heterogeneous postdispersal process. Klein, Carpentier, and Oddou‐Muratorio (2011) showed that Bayesian estimates with individual random fecundities were more robust estimates than maximum likelihood ones. Additionally, we compared the effective reproductive success (RS) as estimated with MEMM model and the realized reproductive success estimated with a categorical parentage analysis (CERVUS, Marshall, Slate, Kruuk, & Pemberton, 1998). There is a clear correlation between both effective and realized reproductive success, and outlier points correspond to cases where discrepancies are indeed expected due to peculiar spatial location of trees (Supporting information S4.2).

### 2.5 Phenotypic selection gradients and selection differentials

We used regression‐based approaches to estimate univariate linear (β*_x_*) and quadratic (γ*_x_*) selection gradients at generation G1 according to the following linear model:

(1) $$w=\mu +C+E+P+\beta_{x}x+\left(\frac{\gamma x}{2}\right)x^{2}+\varepsilon$$

where *w* is the relative fitness (relative value of RS), *µ* is the population mean, *β_x_* is the linear univariate selection gradient and *γ_x_* the quadratic selection gradient, and *x* is the trait value expressed as a standardized value (standardized according to the standard deviation).*C*, *E* and *P* are covariables accounting for the ecological and environmental conditions close to each G1 tree potentially correlated with fitness or to fitness components:

*C* is a competition index (Hegyi index [Hegyi, 1974]) measuring the local density around each tree. C of tree *j* is calculated as follows:

(2) $$C_{j}=\sum_{i=1}^{n}\frac{D_{i}}{D_{j}{\text{Dist}}_{\mathrm{ij}}}$$

where *n* is the number of trees within the neighbourhood of the subject tree *j* (the neighbourhood of the subject tree is a circle of radius 10 m), *D_j_* is the diameter at breast height of the subject tree *j*, *D_i_* is the diameter at breast height of tree *i* standing in the neighbourhood of the subject tree, and Dist*_ij_* is the distance between subject tree *j* and tree *i*.

*E* is an environmental index combining ecological variables downscaled to the level of each G1 and G2 tree (altitude, pH, soil moisture, carbon‐to‐nitrogen ratio and organic matter content) (Alexandre et al., 2020; Truffaut et al., 2017). Because these variables were correlated, a principal component analysis was performed with these variables, and *E* is the value of the first principal component.

*P* is a spatial index that accounts for the spatial autocorrelation of fitness values potentially generated by the spatial structure of tree populations, contributing to the nonindependence of residuals in model 1. We used the PCNM method (principal coordinates of neighbour matrices) suggested by Marrot, Garant, and Charmantier (2015) and based on Borcard and Legendre (2002) to account for this autocorrelation. PCNM extracts eigenvectors from a distance matrix describing the spatial structure of the data. PCNM was implemented from a distance matrix including all the individuals in the area, separately for each species, with a threshold value *t* equal to the maximum distance between two trees in the plot. The PCNM analysis generated multiple eigenvectors. For each eigenvector, we ran a linear regression of fitness analysis (model 2). The AIC (Akaike information criterion) values for the linear regressions were compared, and the eigenvector minimizing AIC was selected as a covariate for subsequent models. Unlike Marrot et al. (2015), we chose to include only one eigenvector, to prevent problems of overparametrization, and this proved to be sufficient to eliminate the spatial autocorrelation in fitness.

As the calculation of *β_x_* is based on standardized values of *x*, *β_x_* is equivalent to the intensity of selection (or differential selection expressed in standard deviation units) operating on generation G1, the number of standard deviations by which selection in G1 shifts the mean values of trait *x* (Matsumura, Arlinghaus, & Dieckmann, 2012).

(3) $$\beta_{x}=x^{*}‐x$$

where *x* ^*^ and *x* are the mean trait values of G1 trees after and before selection, respectively.

### 2.6 Response to selection

#### 2.6.1 Predicted responses

We used the second theorem of selection (STS) (Price, 1970; Robertson, 1966) to predict the evolutionary response to selection (*R_e_*), by estimating the additive covariance between relative fitness (*w_a_*) and the trait of interest (*x_a_*):

(4) $$R_{e}=\text{Cov}\left(w_{a},x_{a}\right)$$

By simultaneously estimating the additive covariance and variance of relative fitness and the trait, this approach overcomes the inflating bias of *R_e_* potentially due to the correlation of environmental effects when the breeder's equation is used to predict *R_e_*. This method provides an overall estimate of the expected genetic shift of the trait, whether due to direct or indirect selection via other traits or to other evolutionary forces (Morrissey et al., 2012; Stinchcombe et al., 2014).

We used bivariate animal models to estimate the additive covariance between relative fitness and the trait, in the R package breedR (Munoz & Sanchez, 2018).

(5) $$\left[\begin{array}{c} w\\ x \end{array}\right]=\mu +Y_{1}c+Y_{2}e+Y_{3}p+Za+\varepsilon$$

where ***w*** is the subvector of relative fitness and ***x*** is the subvector of the trait of interest, **c** is the vector of the fixed effect of competition, **e** the vector of the fixed effect of environment, **p** the vector of the fixed spatial effect as they are defined in (1), **a** is the vector of random genetic (additive) effect of *w* and *x*, **ε** is the vector of the random effect of the residuals, and Y_1_, Y_2_, Y_3_ and Z are the index matrices related to each effect (Alexandre et al., 2020).

The parameter we wish to estimate in (5) is the genetic covariance between fitness (*w*) and the trait (*x*), given the bivariate normal distribution assumed for the genetic values in the animal model:

(6) $$a=\left[\begin{array}{c} w_{a}\\ x_{a} \end{array}\right]∼\left(0,\left[\begin{array}{cc} G\;\text{Var}\left(w_{a}\right) & G\text{Cov}\left(w_{a},x_{a}\right)\\ G\text{Cov}\left(w_{a},x_{a}\right) & G\text{Var}\left(x_{a}\right) \end{array}\right]\right)$$

where ***G*** is the additive genetic relationship matrix between the trees of generation G1. We used the realized genomic relatedness to estimate the components of ***G***. In a previous publication (Alexandre et al., 2020; Lesur et al., 2018) retrieved a large number of SNPs from a genomic capture analysis, making it possible to construct the genomic relatedness matrix (***G***) between any pair of G1 trees as follows:

(7) $$G=\frac{\left(M‐P\right){\left(M‐P\right)}^{t}}{2\sum \left(1‐p_{i}\right)p_{i}}$$

where M is an n*m matrix (*n* being the number of G1 individuals and *m* the number of SNPs) of genotypic arrays scored as −1, 0 or 1 for homozygote, heterozygote and alternative homozygote, respectively, P is an n*m matrix of allele frequencies computed as 2(*p*_i_−0.5), and *p_i_* is the frequency of the second allele (alternate to the minor allele) at locus *i*, as described by (VanRaden, 2008), determined with the kin function of the R package synbreed (Wimmer, Albrecht, Auinger, & Schon, 2012). The variance of relatedness was higher in *Q. petraea* (0.0025) than in *Q. robur* (0.0011), and the total number of SNPs used to retrieve realized genomic relatedness was 15,274 in *Q. petraea* and 16,408 in *Q. robur* (Alexandre et al., 2020). The variance of realized genomic relatedness, which is crucial for the estimation of genetic variance–covariances with the animal model (Walsh & Lynch, 2018 p.692), was similar to that reported for other outbred species (Csillery et al., 2006; Perrier, Delahaie, & Charmantier, 2018).

BreedR is a linear mixed model (LMM) based on restricted maximum likelihood estimation. GLMMs (generalized linear mixed models) are not available in this package. We therefore used LMMs only. For fitness and/or traits not following a Gaussian distribution, we proceeded as follows: (a) the data were log‐transformed, (b) the bivariate LMM was fitted to the transformed data and used to predict evolutionary changes on the transformed scale, and (c) the QGglmm package (de Villemereuil, 2018) was used to obtain variance–covariance matrices and to predict changes at the scale of the untransformed data. We present variances and covariances with their standard errors on the transformed scale and phenotypic predictions at the scale of the untransformed data.

#### 2.6.2 Observed responses

We tried to compare the responses predicted with STS and the realized responses within generation G2. However, this proved challenging as obvious biological constraints made it impossible to set up a common garden of G1 and G2 plants. We adopted two different methods for assessment of the observed responses in the next generation and compare the observed responses with those predicted by STS. We first investigated whether there was a shift at the genetic level, by estimating breeding values in both generations from the animal model described in (5) implemented in the univariate context and with the addition of generation as a fixed term (Walsh & Lynch, 2018 p. 706).

(8) $$X=\mu +Y_{1}c+Y_{2}e+Y_{3}p+Y_{4}g+Za+I\varepsilon$$

Using model (8), we calculated the breeding values of all trees of generations 1 and 2 and their mean values/generation were compared for all traits assessed in both generations. The relatedness matrix G of (8) differs from that of model 5 by including the genetic relatedness between trees of G1, between trees of G1 and G2 and between trees of G2. It therefore combines realized genomic relatedness and the relatedness inferred from the parentage analysis between G1 and G2, as described by Alexandre et al. (2020). Hence, sib–sib relationships in G2 and parent–offspring relationships between G1 and G2 trees were inferred from the parentage analysis based on 82 SNPs (Truffaut et al., 2017). In a previous companion paper, we showed that realized genomic relatedness assessed in a subsample of G2 half sibs and full sibs matched the pedigree relatedness (Lesur et al., 2018), thus allowing to construct the G matrix combining genomic relatedness and pedigree inferred relatedness.

In addition to comparing breeding values, we also used a more empirical method to assess generational shifts at the phenotypic level. We compared the phenotypic means of traits assessed in G2 trees, according to the fitness value of their parents in G1. Indeed, pedigree relationships between the two generations were reconstructed in a previous study (Truffaut et al., 2017) making it possible to segregate offspring on the basis of the relative fitness of their parents.

We subdivided the G1 trees into two categories: the top 50% in terms of observed fitness and the bottom 50% in terms of observed fitness. We then screened the offspring in the two classes on the basis of their pedigree relationships. Finally, we compared the phenotypic values of the G2 trees between the two categories.

Comparisons of phenotypic and genetic differences between the two categories were performed for the 11 traits assessed in G2 trees that were also assessed in G1 trees. Ultimately, we were therefore able to compare the genetic expected response (Re) based on the genetic covariance between the traits and fitness of G1 trees with the realized genetic and phenotypic shifts in their G2 offspring. However, comparisons of absolute values would be meaningless, as the two approaches are based on different methods. These comparisons should therefore be seen as a qualitative empirical attempt to compare predicted and realized evolutionary changes. We thus limited our observations to comparisons of the signs of the shifts provided by the two methods.

## 3 RESULTS

### 3.1 Distribution and variation of fitness

**FIGURE 1:**
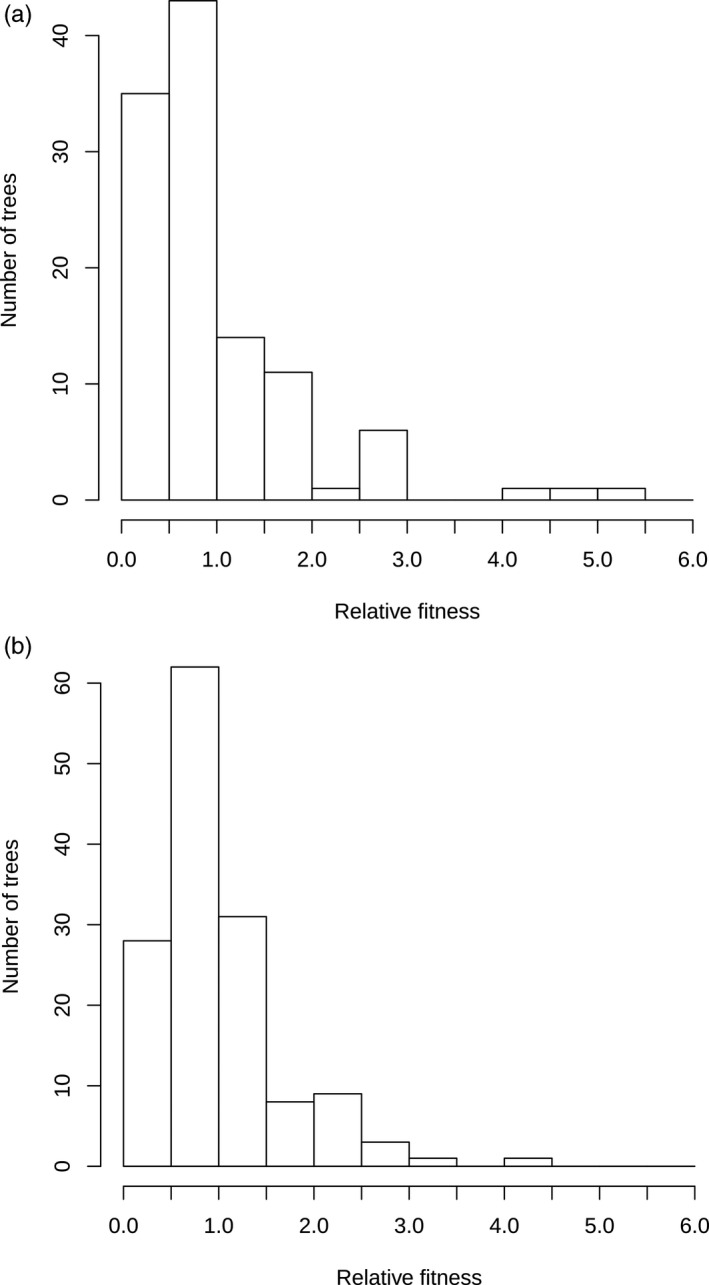
(a) Distribution of relative fitness in *Quercus petraea*. (b) Distribution of relative fitness in *Q. robur*

Fitness, measured as reproductive success at the age of 20 years and expressed as relative fitness, had a skewed distribution within each species, with higher densities at lower values, particularly for *Q. petraea* (Figure 1). The variance of relative fitness, also known as the opportunity for selection (I, [Endler, 1986]), was higher in *Q. petraea* than in *Q. robur* for RS (0.611 vs. 0.420). When the univariate animal model was used with log‐transformed relative fitness data to subdivide the overall phenotypic variance into genetic and residual terms (univariate model 5), additive genetic variance was found to be greater in *Q. petraea* than in *Q. robur* (0.468 vs. 0.193) (Table 2). Finally, the sampling variance of the genetic variance was also higher in *Q. robur*, to the extent that the confidence interval encompassed 0. Below, we log‐transform the fitness data for the estimation of phenotypic gradients and genetic covariances with the different traits.

**TABLE 2:** Variance components of the recruitment success (RS)

Species	Overall variance	Genetic variance (Va)	SE (Va)
*Quercus petraea*	0.611	0.468	0.105
*Quercus robur*	0.420	0.193	0.168

### 3.2 Phenotypic selection gradients

In both species, significant linear selection gradients were found for radial growth‐related traits (CIRC, RSURF, RWDTH) (Table 3), suggesting that selection favours trees with better growth. However, selection trends differed between the species for other traits. In *Q. petraea*, significant β values were obtained only for leaf nitrogen content (N) and three metabolites (MVL, PNTL and VSCL). In *Q. robur*, linear gradients were significant for leaf morphological traits (BS, HR, PL, PR) and one secondary metabolite (TWK).

**TABLE 3:** Linear phenotypic selection gradients in *Quercus petraea* and *Q. robur*

		*Quercus petraea*		*Quercus robur*	
	Trait	β	*p*val(β)	β	*p*val(β)
Growth	CIRC	0.371	1.6 × 10^–3^	**0.221**	5 × 10^–4^
	HGHT	0.129	.17	0.118	.06
	RSURF	**0.419**	2.2 × 10^–4^	**0.214**	6.2 × 10^–4^
	RWDTH	**0.408**	2.7 × 10^–4^	**0.217**	5.9 × 10^–4^
Phenology	LU	−0.039	.95	0.078	.27
	LS	−0.238	.07	0.088	.10
	GSL	−0.110	.33	−0.039	.72
	FFLW	0.005	.37	−0.002	.73
	MFLW	0.075	.61	0.008	.78
	MAR	−0.121	.27	0.051	.19
Physiology	C	−0.329	.06	0.056	.69
	C/*N*	0.079	.19	−0.013	.78
	δ^13^C	0.009	.71	0.043	.21
	δ^15^N	−0.010	.74	−0.041	.83
	MLA	0.020	.59	−0.011	.95
	*N*	**−0.245**	.04	0.052	.36
	SLA	−0.032	.62	−0.015	.99
Resilience	REC	0.030	.57	−0.035	.24
	REL	0.019	.34	−0.075	.24
	RET	−0.044	.72	−0.036	.71
Structure	WD	−0.166	.17	0.064	.18
Leaf morphology	BS	−0.033	.31	**0.092**	.01
	HR	0.163	.08	**−0.097**	.04
	LDR	−0.093	.31	0.113	.14
	LL	−0.064	.55	−0.040	.42
	LW	−0.063	.38	0.001	.65
	LWR	−0.018	.44	0.081	.63
	NL	−0.032	.88	−0.105	.15
	NV	−0.123	.15	0.036	.76
	OB	0.099	.65	−0.013	.74
	PL	−0.005	.60	**−0.155**	3.3 × 10^–3^
	PR	0.030	.29	**−0.143**	3.8 × 10^–3^
	PV	−0.124	.11	0.058	.51
	SW	−0.043	.54	−0.087	.09
	WP	−0.018	.74	−0.042	.38
Defence	CNFL	−0.172	.09	−0.053	.53
	CSTG	−0.019	.96	−0.022	.82
	CSTL	0.035	.72	0.069	.45
	CWSK	−0.176	.14	−0.003	.78
	EGNL	−0.063	.97	−0.100	.09
	ELAC	−0.091	.75	0.040	.25
	ELTOT	−0.014	.71	0.095	.15
	GRDN	−0.127	.38	−0.016	.76
	MVL	**0.258**	.02	−0.019	.83
	PNTL	**0.151**	.05	−0.072	.20
	ROBA	−0.101	.13	−0.028	.54
	ROBB	−0.148	.39	−0.007	.91
	ROBC	0.036	.86	0.006	.83
	ROBD	0.082	.53	0.035	.57
	ROBE	−0.108	.35	−0.035	.60
	SYRG	−0.105	.35	−0.092	.13
	TWSK	0.023	.53	**−0.101**	.04
	VNL	−0.144	.30	−0.117	.08
	VSCG	0.080	.75	−0.015	.99
	VSCL	**0.234**	.01	0.078	.36
	X2PHL	−0.103	.66	−0.056	.57

Note

β: selection gradient, selection differential. *p*val(β) is the *p* value regarding the statistical test of the linear regression coefficient in model 1.

Bold values correspond to selection gradients exhibiting p < 0.05.

Phenotypic quadratic selection gradients were significant only for C/N in *Quercus robur* (Supporting information S6).

### 3.3 Genetic covariances between fitness and traits

Mixed animal model estimates highlighted significant genetic covariances (Table 4). In *Q. petraea*, genetic covariances were significant for growth, radial and vertical growth, physiological traits (C, MLA and SLA), leaf morphological traits (NL, OB, PR) and several secondary metabolites, including ellagitannins and volatile compounds.

**TABLE 4:** Genetic covariances between the traits and relative fitness

Trait		*Quercus petraea*			*Quercus robur*		
Category	Trait	Cov_a_	St.Cov_a_	SE.Cov_a_	Cov_a_	St.Cov_a_	SE.Cov_a_
Growth	CIRC	11.694*	0.398	3.858	1.175	0.053	5.513
	HGHT	48.392*	0.325	24.040	−38.943*	−0.245	7.162
	RSURF	**380.980**	0.461	185.390	−102.450*	−0.183	15.991
	RWDTH	0.194*	0.461	0.043	−0.061	−0.177	0.013
Phenology	LU	0.113	0.025	0.370	−0.471	−0.081	0.738
	LS	−0.651	−0.166	0.343	1.083	0.288	0.695
	GSL	−0.574	−0.103	0.474	1.516	0.244	0.887
	FFLW	0.646	0.098	0.678	0.513	0.103	1.044
	MFLW	0.282	0.054	0.409	NA	NA	NA
	MAR	−0.299	−0.199	0.269	0.274	0.163	0.356
Physiology	C	−12.937*	−0.430	2.038	0.253	0.032	1.893
	C.*N*	0.273	0.076	0.343	0.173	0.047	0.169
	d13C	0.048	0.043	0.145	0.074	0.073	0.073
	d15N	0.108	0.079	0.214	0.093	0.081	0.254
	MLA	**2.681**	0.265	0.792	−2.843*	−0.287	0.424
	*N*	−0.459	−0.182	0.234	NA	NA	NA
	SLA	**−0.501**	−0.215	0.216	NA	NA	NA
Resilience	REC	0.033	0.221	0.021	0.023	0.065	0.076
	REL	0.006	0.068	0.007	−0.015	−0.070	0.045
	RET	−0.006	−0.080	0.010	−0.018	−0.150	0.023
Structure	WD	−7.981	−0.277	4.520	−5.338	−0.199	4.259
Leaf morphology	BS	−0.074	−0.094	0.060	0.210*	0.176	0.090
	HR	−0.005	−0.006	0.126	−0.202	−0.315	0.117
	LDR	−0.015	−0.003	0.681	NA	NA	NA
	LL	−1.388	−0.087	1.774	2.851	0.204	2.781
	LW	−0.760	−0.124	0.810	0.776	0.160	0.636
	LWR	−0.483	−0.139	0.500	−0.094	−0.033	0.623
	NL	0.423*	0.315	0.077	0.306	0.235	0.167
	NV	−0.012	−0.021	0.095	0.235	0.171	0.182
	OB	**0.748**	0.146	0.124	NA	NA	NA
	PL	0.226	0.046	0.226	−0.253	−0.115	0.484
	PR	**0.312**	0.102	0.150	−0.689	−0.307	0.505
	PV	−0.617	−0.100	1.216	2.348	0.146	1.740
	SW	−0.476	−0.106	0.658	0.275	0.094	0.576
	WP	−0.091	−0.009	2.160	1.277	0.145	1.223
Defence	CNFL	−0.250*	−0.432	0.038	−0.147	−0.199	0.107
	CSTG	**0.912**	0.184	0.373	−0.699	−0.157	1.015
	CSTL	0.048*	0.089	0.007	0.079	0.153	0.063
	CWSK	−0.546	−0.341	0.377	−0.248	−0.235	0.152
	EGNL	−0.464*	−0.371	0.076	−0.019	−0.016	0.101
	ELAC	−0.111	−0.207	0.088	−0.016	−0.036	0.091
	ELTOT	−0.052	−0.115	0.008	0.068	0.163	0.061
	GRDN	0.107	0.036	0.441	−0.213	−0.075	0.621
	MVL	0.038*	0.059	0.006	0.040	0.058	0.148
	PNTL	0.008	0.014	0.121	−0.147	−0.212	0.111
	ROBA	−0.184	−0.120	0.174	0.342	0.135	0.257
	ROBB	**−0.706**	−0.297	0.316	0.517	0.256	0.278
	ROBC	**−0.104**	−0.258	0.031	0.000	−0.001	0.095
	ROBD	0.141	0.041	0.490	−0.163	−0.046	0.691
	ROBE	−0.376	−0.160	0.453	−0.514	−0.286	0.370
	SYRG	−0.342	−0.134	0.675	NA	NA	NA
	TWSK	0.033	0.020	0.306	−0.325	−0.305	0.133
	VNL	**−0.163**	−0.322	0.046	−0.065	−0.092	0.073
	VSCG	0.423	0.087	0.257	−0.178	−0.037	1.017
	VSCL	**0.160**	0.240	0.070	0.082	0.138	0.127
	X2PHL	**−0.216**	−0.328	0.042	0.040	0.049	0.172

Note

Bold values indicate significant genetic covariances. Asterisks indicate significant genetic variances of the trait.

Abbreviations: Cov_a_, genetic covariance between the trait and relative fitness; St.Cov_a_, standardized genetic covariance (= Cov_a_/σ_p_, with σ_p_ = phenotypic standard deviation); SE.Cov_a_, standard error of the genetic covariance.

In *Q. robur*, the genetic covariances calculated between fitness and traits (Table 4) must be interpreted with caution because the genetic variance of fitness was not significantly different from 0 (Table 2). This may also have contributed to convergence difficulties for the estimation of var/cov for a few traits (MFLW, N, SLA, LDR, OB, SYRG). Only growth traits (HGHT, RSURF), MLA and basal shape of leaf lamina (BS) presented significant covariance with fitness (Table 4).

As we focused on the qualitative evolutionary response of the traits (i.e. the sign of the covariance), we compared the sign of the selection gradients (Table 3) and the sign of the genetic covariance (Table 4). Formally, the covariance of the numerator of the selection gradient can be broken down as follows:

(9) $$\text{Cov}\left[w,X\right]=\text{Cov}\left(w_{a},X_{a}\right)+\text{Cov}\left(w_{e},X_{a}\right)+\text{Cov}\left(w_{a},X_{e}\right)+\text{Cov}\left(w_{e},X_{e}\right)$$

where ***W**_e_* and ***X**_e_* are the environmental values associated with relative fitness and the targeted trait.

There is no biological support for $\text{Cov}\left(w_{e},X_{a}\right)$ and $\text{Cov}\;\left(w_{a},X_{e}\right)$ being non‐negligible terms in 9. Hence, discrepancies between the signs of the selection gradient and the genetic covariances are probably due to $\text{Cov}\left(w_{a},X_{a}\right)$ and $\text{Cov}\left(w_{e},X_{e}\right)$ having opposite signs. This might be the case for growth components in *Q. robur* with positive selection gradients but negative genetic covariances. A similar interpretation may explain why more traits in *Q. petraea* have significant genetic covariances than significant selection gradients.

### 3.4 Predicted versus observed selection responses

**FIGURE 2:**
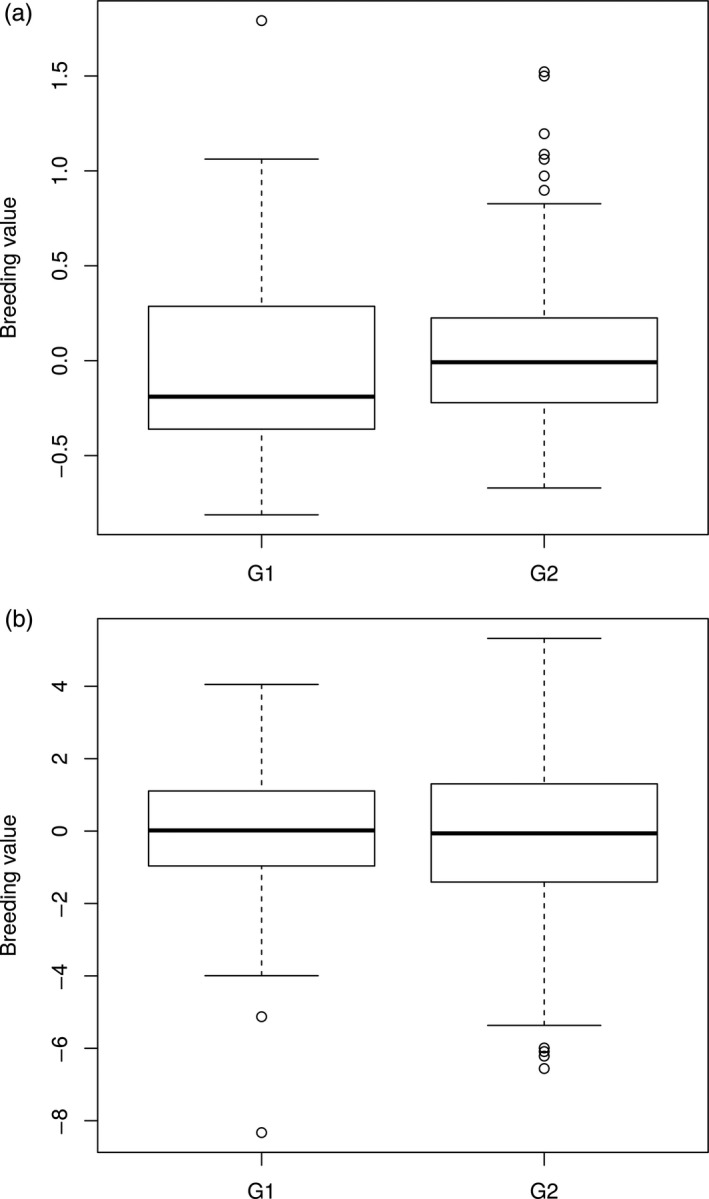
(a) Box plot of breeding values of *Quercus petraea* for circumference in generation 1 (G1) and generation 2 (G2). Breeding values are standardized across the two generations and have only a scaling value, rather than a biological meaning. (b) Box plot of breeding values of *Quercus petraea* for height in generation 1 (G1) and generation 2 (G2). Breeding values are standardized across the two generations and have only a scaling value, rather than a biological meaning

**FIGURE 3:**
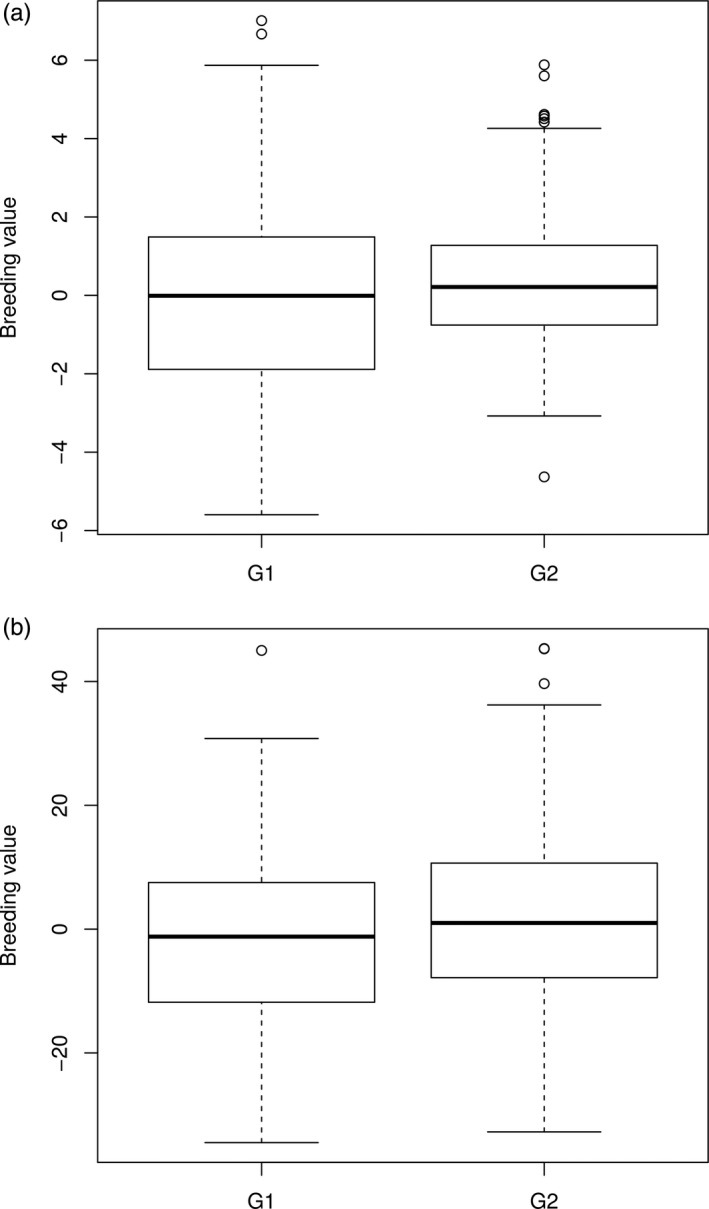
(a) Box plot of breeding values of *Quercus robur* for circumference in generation 1 (G1) and generation 2 (G2). Breeding values are standardized across the two generations and have only a scaling value, rather than a biological meaning. (b) Box plot of breeding values of *Q. robur* for height in generation 1 (G1) and generation 2 (G2). Breeding values are standardized across the two generations and have only a scaling value, rather than a biological meaning

We compared mean breeding values between G1 and G2 trees for the subset of traits assessed in both generations (HGHT and CIRC) and for which model 8 could be applied, that is traits of interest and covariates (for fixed effects) available for both G1 and G2 trees (see Table 1). In model 8, we accounted for changes in environmental conditions between the two generations and for differences in the age at which traits were measured in the two generations, by introducing a fixed effect of generation into the model. We recognize that this may result in some of the genetic differences between the two generations being absorbed into the fixed effect of generation in model 8, resulting in an underestimation of the genetic changes between generations. Genetic shifts were, however, observed in the mean values of the two traits in *Q. petraea* (Figure 2), whereas only limited changes were seen for *Q. robur* (Figure 3). These results are consistent with the genetic shifts predicted by STS exclusively from assessments made in G1, with the exception of HGHT in *Q. robur* (Table 4).

In addition to measuring shifts at the genetic level, we also attempted to assess changes at the phenotypic level, by an empirical method. It was impossible to compare phenotypes of trees of the two generations at the same age experimentally or to raise a control population to which the phenotypes of each generation could be compared. As pedigree relationships were reconstructed by parentage analysis, G2 saplings were separated in two subsets defined on the basis of parental fitness (G2[+] saplings, the parents of which had the highest RS values, and G2[−] saplings, the parents of which had the lowest RS values; see methods). This made it possible to make empirical qualitative comparisons between mean phenotypic values in G2 saplings according to the fitness of their parents (Tables 5 and 6).

**TABLE 5:** Comparison between predicted and observed selection responses in *Quercus petraea*

Trait Class	Trait^a^	Trait units	Predicted genetic responses^b^			Observed phenotypic responses^c^		
			G1	G2	Re %	G2 −	G2 +	Δ(%)
Growth	CIRC*	cm	190.83	200.94	5.30	32.43	33.52	3.32
	HGHT*	cm	2,655.22	2,696.84	1.57	1,089.00	1,106.573	1.60
Phenology	LU	Nb of days	106.21	106.31	0.095	3.92^d^	3.92^d^	0.13^d^
Physiology	C*	g/kg	454.74	443.51	−2.47	458.69	458.178	−0.11
	C/N	ratio	24.53	24.77	0.99	19.29	19.132	−0.83
	δ^13^C	‰	−29.77	−29.73	0.14	−29.46	−29.59	0.44
	δ^15^N	‰	−3.35	−3.25	2.87	−6.42	−6.46	0.65
	**MLA**	cm^2^	44.78	47.13	5.25	32.21	32.51	0.92
	N	g/kg	18.84	18.44	−2.17	24.03	24.14	0.47
	**SLA**	m^2^/kg	11.93	11.48	−3.76	13.21	13.49	2.12
Structure	WD	kg/m^3^	576.77	569.87	−1.20	506.97	509.31	0.46

^a^ Traits in bold characters exhibit significant genetic covariances with fitness in G1 (Table 4). The asterisk indicates that the trait exhibits also significant genetic variance in G1.

^b^ G1 is the phenotypic mean of G1 trees. G2 is the predicted mean of G2 and Re % is the predicted response using STS (Second Theorem of Selection; Price, 1970; Robertson, 1966). Re% = (G2−G1) × 100/G1.

^c^ Observed phenotypic response (Δ%) corresponds to the difference between the phenotypic mean values of the offspring of parent trees with higher observed fitness (G2+) and lower observed fitness (G2−), relative to the mean of (G2+) and (G2−).

^d^ LU is assessed by as score of bud development in generation 2 (three last columns). The positive sign of the shift (Δ% = 0.13) indicates a shift towards earlier flushing times.

**TABLE 6:** Comparison between predicted and observed selection responses in *Quercus robur*

Trait class	Trait^a^	Trait units	Predicted genetic responses^b^			Observed phenotypic responses^c^		
			G1	G2	Re %	G2−	G2 +	Δ(%)
Growth	CIRC	cm	167.19	168.47	0.76	26.695	27.455	2.81
	HGHT*	cm	2,516.41	2,474.53	−1.66	924.252	945.46	2.27
Phenology	LU	Nb of days	101.80	101.28	−0.50	3.687^d^	3.685^d^	−0.05^d^
Physiology	C	g/kg	461.31	461.59	0.06	465.434	465.098	−0.07
	C/N	ratio	23.69	23.88	0.80	20.205	19.991	−1.06
	δ^13^C	‰	−30.08	−30.00	0.27	−29.657	−29.713	−0.19
	δ^15^N	‰	−2.32	−2.22	4.39	−3.904	−4.156	−6.25
	MLA*	cm^2^	35.78	32.72	−8.53	22.773	23.351	2.51
	*N*	g/kg	19.84	NA	NA	23.362	23.579	0.92
	SLA	m^2^/kg	13.82	NA	NA	13.49	13.866	2.75
Structure	WD	kg/m^3^	549.69	543.78	−1.07	498.269	497.628	−0.13

^a^ Traits in bold characters exhibit significant genetic covariances with fitness in G1 (Table 4). The asterisk indicates that the trait exhibits also significant genetic variance in G1.

^b^ G1 is the phenotypic mean of G1 trees. G2 is the predicted mean of G2 and Re % is the predicted response using STS (Second Theorem of Selection, Price, 1970; Robertson, 1966). Re% = (G2−G1) × 100/G1.

^c^ Observed phenotypic response (Δ%) corresponds to the difference between the phenotypic mean values of the offspring of parent trees with higher observed fitness (G2+) and lower observed fitness (G2−), relative to the mean of (G2+) and (G2−).

^d^ LU is assessed by as score of bud development in generation 2 (three last columns). The negative sign of the shift (Δ% = −0.05) indicates a shift towards later flushing times.

For *Q. petraea*, convergence between predicted genetic and observed phenotypic responses was observed for traits with significant genetic variance and covariance (CIRC, HGHT and C) (Table 5).

For *Q. robur*, no consistent pattern emerged for the relationship between predicted genetic and observed phenotypic responses. Conflicting results were even obtained for HGHT, for which STS predicted a significant decrease, whereas a phenotypic increase was observed (Table 6). However, both genetic (Figure 3b) and phenotypic (Table 6) responses indicated that HGHT increased from G1 to G2.

Overall observed responses (either by estimating breeding values or using the empirical method) were more in agreement with predicted responses for *Q. petraea* than for *Q. robur*, These species differences should be related to the convergence constraints observed for the estimation of genetic variance/covariances for *Q. robur* (Table 4).

## 4 DISCUSSION

We screened numerous oak phenotypic traits for the expected genetic changes over two generations in an oak forest subject to global environmental changes and even‐aged silvicultural regimes. Despite the short time period covered by this study, we detected evolutionary patterns concerning traits likely to change and traits likely to be unaffected by contemporary selection pressures. In both species, growth, leaf morphology and physiology, and defence‐related traits displayed significant selection differentials and predicted shifts, whereas phenology, water metabolism, structure and resilience‐related traits did not. For a few traits, mostly related to growth, we were able to compare predicted and observed responses in the next generation at the genetic and phenotypic levels. The results obtained suggest that this approach may provide valuable insights into ongoing genetic trends in oak forests, despite technical and biological constraints. We discuss these results in the light of the experimental limitations of this study and the differential demographic dynamics of *Q. petraea* and *Q. robur* in response to ongoing environmental changes (Truffaut et al., 2017). We reiterate here that our approach emphasized mainly the qualitative shifts observed (direction) and did not allow to identify the likely underlying causes of the evolutionary shifts, whether they were the direct response of selection, or the indirect response via selection on other traits, or due to any other evolutionary forcing factor. However, our data resources may allow future investigations aiming at disentangling the underlying evolutionary drivers and direct targets of selection as proposed by Walsh and Lynch (2020, p 682–686).

### 4.1 Traits responding to contemporary selection

Both *Q. petraea* and *Q. robur* displayed strong positive selection gradients for growth‐related traits. This pattern was expected, as competition for light during the early years and subsequent thinning operations tend to eliminate slower growing seedlings and trees. Here, both natural selection and human‐mediated intervention enhance directional selection for faster height growth (Jarret, 2004). Positive gradients for diameter growth may reflect the allometric relationship between primary and secondary growth in trees (del Rio, Bravo‐Oviedo, Ruiz‐Peinado, & Condes, 2019). Leaf morphological traits also responded to selection at either the phenotypic (selection gradients) or the genetic (genetic covariance) level. Interestingly, leaf morphological traits correlated with fitness were species diagnostic traits. In *Q. robur*, the positive correlation of fitness with basal lamina shape (BS) and its negative correlation with hairiness (HR) and petiole length (PL) indicate that selection is operating in favour of *Q. robur*‐like phenotypes (Kremer et al., 2002). Similarly, for *Q. petraea*, the positive correlations of fitness with petiole length (PR) and the number of lobes (NL) indicate that *Q. petraea*‐like trees have a greater fitness, for these traits (Kremer et al., 2002). These results reconcile earlier results indicating that hybrids were more numerous at the seed stage than at the sapling stage (Bacilieri, Ducousso, Petit, & Kremer, 1996; Truffaut et al., 2017), suggesting that hybrid forms tend to be eliminated as the stand ages, resulting in the maintenance of species integrity in populations of sympatric interfertile oak species. Overall, these results suggest that disruptive interspecific selection is driving the two species further apart. It is therefore unlikely that leaf morphological traits are the causal traits targeted by selection. Instead, they probably represent a correlative response of other functional traits involved in species differentiation.

For the more integrative leaf morphological traits, such as SLA and MLA, we observed a selective trend towards larger leaves (MLA, positive predicted genetic responses) and thicker and/or denser leaves (SLA, negative predicted genetic responses) in *Q. petraea*. Together, these two trends suggest that *Q. petraea* is moving towards more efficient photosynthetic capacity (leaves with a lower SLA generally contain more photosynthetic machinery per unit area (Vitousek, Field, & Matson, 1990)) and a larger assimilation area, potentially leading to faster growth. However, negative phenotypic selection gradients were found for nitrogen content (N) in *Q. petraea*, contrary to the trends reported for growth and leaf morphological traits. Indeed, lower leaf nitrogen content generally leads to lower protein contents and, hence, lower leaf maximum photosynthetic capacity (Evans, 1989). This trend towards lower leaf N content may not, however, reflect adaptation. It may instead be a consequence of declining N availability over time in many unfertilized terrestrial ecosystems due to increases in atmospheric carbon dioxide levels and longer growing seasons (Craine et al., 2018). We observed no particular trend for foliar δ^15^N, which is usually positively associated with N availability relative to plant N demand (as plants experiencing lower N availability acquire soil N less rich in ^15^N, (Craine et al., 2015)). By contrast, only one trait displayed significant covariance with fitness in *Q. robur*: a negative genetic response for MLA (trend towards smaller leaves).

The last category of traits displaying genetic shifts concerned secondary compounds present in oak wood. The predicted genetic shifts were significant for 10 of these compounds in *Q. petraea*, whereas no change was predicted for *Q. robur*. Increases in the levels of ellagitannins and other volatile compounds were predicted, whereas decreases were predicted for other metabolites, consistent with a redistribution within the overall mixture in *Q. petraea*. The individual roles of these compounds have not been studied, but their overall biological activity is consistent with reactivity in the insect gut, in which they may cause oxidative stress (Moilanen et al., 2016; Salminen & Karonen, 2011). In American white oaks (*Q. gambelii* × *Q. grisea* hybrid swarm), significant correlations have been found between ellagitannin phenotypes and leaf miner communities (Yarnes, Boecklen, & Salminen, 2008). The synthesis of these compounds has also been reported in the heartwood of oaks and may protect the trunk against pathogen attack (Helm, Ranatunga, & Jervis, 1997). We therefore wonder whether biotic interactions in *Q. petraea* may not have resulted in genetic changes of secondary compounds as was found for example in grasses under selection experiments (Agrawal, Hastings, Johnson, Maron, & Salminen, 2012).

### 4.2 Traits not responding to contemporary selection

Surprisingly, no selective trends were detected for phenological traits in either *Q. petraea* or *Q. robur*. In common garden experiments, considerable genetic differentiation has repeatedly been reported for the date of bud burst, with cogradient genetic variation along temperature gradients (Alberto et al., 2011; Ducousso, Guyon, & Kremer, 1996; Vitasse, Delzon, Bresson, Michalet, & Kremer, 2009). These clines were attributed to adaptive divergence driven by diversifying selection over altitude or latitude gradients (Firmat, Delzon, Louvet, Parmentier, & Kremer, 2017). Furthermore, heritability values are high for the timing of bud flush (Alberto et al., 2011; Baliuckas & Pliura, 2003), as confirmed here, exposing phenology to the possibility of evolutionary shifts. Nevertheless, phenological traits were not correlated with fitness in our study, at either the phenotypic or the genetic level. Two reasons for the observed discrepancy can be proposed. First, phenology may not respond to diversifying selection, and the cogradient genetic clines and high heritability may instead be generated by assortative mating. Indeed, preferential mating between extremely early‐ or late‐flushing trees may contribute to the maintenance of high levels of genetic variation and to the generation of cogradient genetic clines, even in the absence of any selection (Soularue & Kremer, 2012, 2014). Alternatively, the selection pressure on flushing time may have been too weak during the recruitment period to have had a detectable effect. An absence of late frost and/or herbivorous insect damage may have protected the sapling cohort from the effects of strong selection acting on early‐flushing saplings. We checked retrospectively the occurrences of late frost that may have caused damages to the seedling and saplings of G2 and found only two occurrences in 1996 and 2003 and at a moderate level (Supporting information S7). Therefore, a likely interpretation to the lack of phenological shift between G1 and G2 is the absence of selection pressures.

Resilience‐related traits were not significantly correlated with fitness. The resilience components monitored here were the tree ring width response during and after so‐called “negative pointer years,” defined as years with substantial less cambial growth than the previous years (Supporting information S2, and Alexandre et al., 2020). These traits therefore express resistance and resilience to extreme drought events occurring during the lifetime of the trees. Six negative pointer years were recorded in *Q. petraea* and nine in *Quercus robur*, between 1921 and 1996, and two such years occurred during the recruitment period (1990 and 1996). Large annual water deficits were also recorded during these two years (Supporting information S5, Figure S8). We suspect that these pointer years had only a temporary impact on fitness, their effects being overridden by subsequent more favourable years.

Finally, no trend (selection gradient or predicted response) was detected for traits relating to water use efficiency (δ^13^C), despite the occurrence of drought events during the regeneration period (Supporting information S5). We think that this is because water use efficiency is correlated with the ability to maintain growth during periods of moderate drought, whereas the drought resistance of seedlings in natural forests is mostly a question of surviving severe drought conditions (thus, fitness). It would be interesting to evaluate traits relating to hydraulic failure (xylem embolism resistance), to determine whether there has been selection for seedlings with greater embolism resistance.

At this stage, it would be relevant to compare the genetic trends observed in this study with large‐scale phenotypic trends monitored in forest trees currently and over very recent time scales. Such phenotypic trends have repeatedly been found in angiosperms and gymnosperms for growth and phenology. In recent decades, trees have displayed a steady increase in growth, interpreted as a response to higher levels of atmospheric CO_2_ and to atmospheric nitrogen deposits (Maes et al., 2019). Continent‐wide phenological surveys have also shown a convergent trend towards earlier bud flushing in tree species in response to increasing temperatures (Menzel et al., 2006; Vitasse, Delzon, Dufrene, et al., 2009; Vitasse et al., 2011). Our results shed further light on these trends, by showing that genetic shifts are in the same direction as phenotypic trends, at least for growth, and that they can therefore be considered as “cogradient trends” (Pemberton, 2010). However, our results do not support the existence of a genetic contribution, through cogradient or counter‐gradient, to the ongoing phenological trends.

### 4.3 Contrasting adaptive responses of *Q. petraea* and *Q. robur*

Our data provide unprecedented estimates of the genetic variance of relative fitness in long‐lived trees, corresponding to empirical estimates of the evolutionary potential in a single generation, as predicted by Fisher's fundamental theorem of natural selection (Fisher, 1930; Shaw & Shaw, 2014). The empirical estimates obtained lie within the range of values compiled in a recent review (Hendry et al., 2018). The overall range extended from 0 to 0.85, but 89% of the values obtained were below 0.20. We obtained mean values of 0.193 for *Q. robur* and 0.468 for *Q. petraea* (Table 2). A similar large difference between the two species was also observed at the phenotypic level (0.420 and 0.611, respectively). Furthermore, the sampling variance of the genetic variance was higher for *Q. robur* than for *Q. petraea*, raising questions as to whether the estimated variance could maintain significant evolutionary potential in *Q. robur*. As a result, directional selection gradients were lower for growth‐related traits and some predicted evolutionary changes were even negative for *Q. robur*. These results raise concerns about the adaptive response of *Q. robur* to contemporary selection pressures and predict different future dynamics for these two species. In a previous paper in which we compared demographic dynamics over two generations in the same study stand, we found that absolute realized reproductive success was also lower in *Q. robur* than in *Q. petraea* and we highlighted a substantial demographic expansion of *Q. petraea* at the expense of *Q. robur* (Truffaut et al., 2017). From G1 to G2, the area occupied by *Q. robur* decreased from 50% to 33%, and the census population size dropped from 50% to 27% (Truffaut et al., 2017). The current demographic dynamics and predicted adaptive responses based on our findings suggest a continuous decline of *Q. robur*, particularly in mixed stands with *Q. petraea*. In addition to the inherent differences in reproductive success revealed here, *Q. robur* may also face the challenge of competitive exclusion by *Q. petraea*, which has better tolerance to drought and higher temperatures (Arend, Brem, Kuster, & Gunthardt‐Goerg, 2013; Vivin, Aussenac, & Levy, 1993). On a larger scale, contrasting distributions are predicted for different oak species as a result of climate change, raising questions about whether our observations reveal an evolutionary element to these dynamics (Madrigal‐Gonzalez et al., 2017). *Q. petraea* was also recently reported to have greater reproductive success than *Q. robur* in mixed stands in Poland (Sandurska, Ulaszewski, & Burczyk, 2019), suggesting that the expansion of *Q. petraea* at the expense of *Q. robur* is occurring over a much larger geographic scale. The concurrent decline of *Q. robur* and expansion of *Q. petraea* observed over a very short time scale here may ultimately lead to contrasting range retraction and expansion in response to ongoing climate change in many ecological contexts and communities (Lenoir & Svenning, 2015).

### 4.4 Limitations and constraints

This study is one of the first to explore the use of evolutionary quantitative genetics for monitoring genetic changes in forest trees *in natura* (Bontemps et al., 2016; Castellanos et al., 2015). More traditional genetic surveys aiming to estimate genetic parameters have been conducted in controlled progeny experiments, and they highlighted the considerable genetic variation within forest tree populations, suggesting that forest trees have the potential to evolve in response to contemporary environmental changes (Cornelius, 1994; Kremer, 1994). However, these investigations were performed in artificial conditions, using experimental designs recommended for breeding purposes (White, Adams, & Neale, 2007), but unsuitable for extension and application to the prediction of responses to contemporary environmental changes in most existing forests undergoing renewal by natural regeneration. The estimation of genetic parameters in naturally regenerating forests, as in this study, faces technical challenges, which we attempted to address here for the specific case of oaks subject to even‐aged silvicultural management regimes. We identified two major constraints and limitations that may have hampered our approach: the persistence of genetic structures in natural regenerated oak forests, the limited variation of genetic relatedness in wind‐pollinated trees. Spatial genetic structures are generated by successive rounds of natural regeneration, particularly in trees in which seed dispersal is limited, as demonstrated for oaks (Sork, 2016; Streiff et al., 1998). Spatial genetic structures may bias the estimation of genetic variances and breeding values in two ways: they contribute to the covariation of environmental and genetic effects, and they generate autocorrelation between neighbouring trees. The issue of the impact of the nonrandomness of environmental effects has already been raised in natural animal populations (Postma & Charmantier, 2007), but is even more crucial in sessile organisms, such as plants with limited seed dispersal. We explicitly accounted for common environment effects by introducing microenvironmental variates into the mixed linear model as fixed effects (models 5 and 8). Spatial autocorrelation, as already demonstrated in our study population (Bacilieri et al., 1994; Streiff et al., 1998), is also accounted for in the animal model used. Correction for spatial genetic structures can be incorporated into the model, but it is difficult to take the biological constraints imposed by the limited variation of genetic relatedness and low levels of connectedness into account, except by increasing sample size. The precision of genetic variance/covariance in natural populations with only distantly related individuals depends mostly on the variance of relatedness and sample size (Vinkhuyzen, Wray, Yang, Goddard, & Visscher, 2013; Visscher & Goddard, 2015). Thus, it is possible to compensate for low levels of variation of relatedness by increasing sample size. Here, the variance of relatedness was of a magnitude similar to that for other outbred species (Csillery et al., 2006; Perrier et al., 2018), but was much lower for *Q. robur* than for *Q. petraea*, potentially hindering convergence for the estimation of genetic covariances in *Q. robur* (Table 4). These constraints have two consequences for estimates of evolutionary changes. In terms of bias, it is unclear whether correcting for spatial structures, as in this study, helps to reduce over‐ or underestimation. Accounting for microenvironmental variation potentially confounded with genetic variation may lead to some of the genetic variation being missed, thereby contribution to the underestimation of genetic variance/covariances. In terms of precision, our results clearly call for larger sample sizes in studies of forest tree populations. The sampling variances for genetic covariances were high for many traits (Table 4), due to the combined effects of limited variation of relatedness and small sample sizes. We therefore recommend the enlargement of long‐term forest plots established decades ago for the monitoring of genetic diversity and evolutionary processes (gene flow, mating system), to cope with the sampling requirements of explorations of evolutionary changes *in natura*. Notwithstanding these limitations, ours results open up encouraging prospects for the implementation of this approach in forests undergoing natural and human‐mediated selection.

## 5 DATA ARCHIVING STATEMENT

The data used in this study (Phenotypic trait values of G1 and G2 trees and their associated ecological variables, and SNP data used to estimate fitness) are accessible at the EVOLTREE eLab repository at the TreePop database, available at http://treepop.pierroton.inra.fr/. To access data, go to the "Publication data" section at the top of the home page, using pc26_treepop as username and pc26_treepop as password. Sequencing data of the G1 trees to estimate their realized genomic relatedness are available in the NCBI – SRA database under the BioProject PRJNA445867 (https://www.ncbi.nlm.nih.gov/bioproject/PRJNA445867/).

## Supporting information

Supplementary Material
