# Supplementary material for: How does contemporary selection shape oak phenotypes?
Source: Evol Appl. 2020 Aug 14;13(10):2772–90. doi: 10.1111/eva.13082 (PMC7691464; doi:10.1111/eva.13082)
Supplement: Supplementary file 1 — Supplementary Material [file EVA-13-2772-s001.docx]

**Supplemental material**

**Supplemental material S1.**

**Natural and human-mediated selection in even-aged oak forests**

The attached figure (Figure S1) illustrates the different steps in the rotation of an oak stand managed under an even-aged silvicultural regime, as practiced in most European countries (Jarret, 2004).

Step 1. Seed cut. When the oak forest stand reaches an appropriate age for renewal, ranging between 80 and 250 years, depending on the site and the species, a seed cut is implemented to reduce tree density to about 100 trees per ha. The seed cut is designed to open up the canopy so that open-pollinated seeds can germinate and produce seedlings. The remaining trees (the seed trees) mate freely, by random mating. They may, of course, be pollinated by pollen from outside the target stand, from nearby oak forests. Oaks are monoecious, allogamous, wind-pollinated species. Self-pollination rates are extremely low, at less than a few percent (Bacilieri, Ducousso, Petit, & Kremer, 1996). The seed cut starts the regeneration phase. In the study stand of La Petite Charnie, the seed cut was performed when the stand was 90-100 years old.

Step 2. Regeneration phase. Depending on fruiting success, the seed trees may be maintained over a number of successive years, through multiple fruiting seasons, to ensure an even distribution of seedlings. Occasionally, additional seed trees may be cut down in areas in which sufficient seed production has occurred. In most cases, the regeneration phase lasts 10 to 12 years. A final cut of all the seed trees is performed when the distribution of seedlings on the ground is even. This marks the end of the regeneration phase. In the study stand of La Petite Charnie, the regeneration phase lasted 11 years. The new G2 generation therefore resulted from mating events over 11 successive years.

Step 3. Seedling phase**.** The cohort of seedlings is allowed to grow, at very high densities, which may exceed 100000 seedlings/ha. The seedling cohort is subject to natural selection driven by competition for resources (light, water, minerals) and/or by biotic interactions (insect herbivory, pathogens). The seedling phase lasts about 10 years, during which time no silvicultural operations are conducted.

Step 4. Sapling phase. Natural selection continues to operate, reducing densities to 2000 to 4000 plants/ha. Thus, during the seedling and sapling phases, more than 90% of the seedlings/saplings are eliminated by natural selection and stochastic mortality. Systematic cleaning operations, in which all saplings located in linear rows of 2-3 m every 6 meters are eliminated, can be implemented occasionally, but such operations do not exert directional selection. In the study stand of the Petite Charnie, phenotypic assessments and parentage analysis were performed at the end of the sapling stage.

Step 5. High-forest phase. This phase is the longest, lasting from a stand age of about 20 to 25 years until the next seed cut. It is characterized by artificial thinning operations at 10-year intervals. Thinnings involve the preferential elimination of trees dominated in the canopy by neighboring trees, or trees displaying disease or insect damage, or with crooked stems. About 100 to 200 trees per ha are removed in each thinning operation. The final thinning operation constitutes the seed cut for the next renewal stage (step 6).

**Figure S1 Illustration of demographic dynamics in even-aged oak forests**


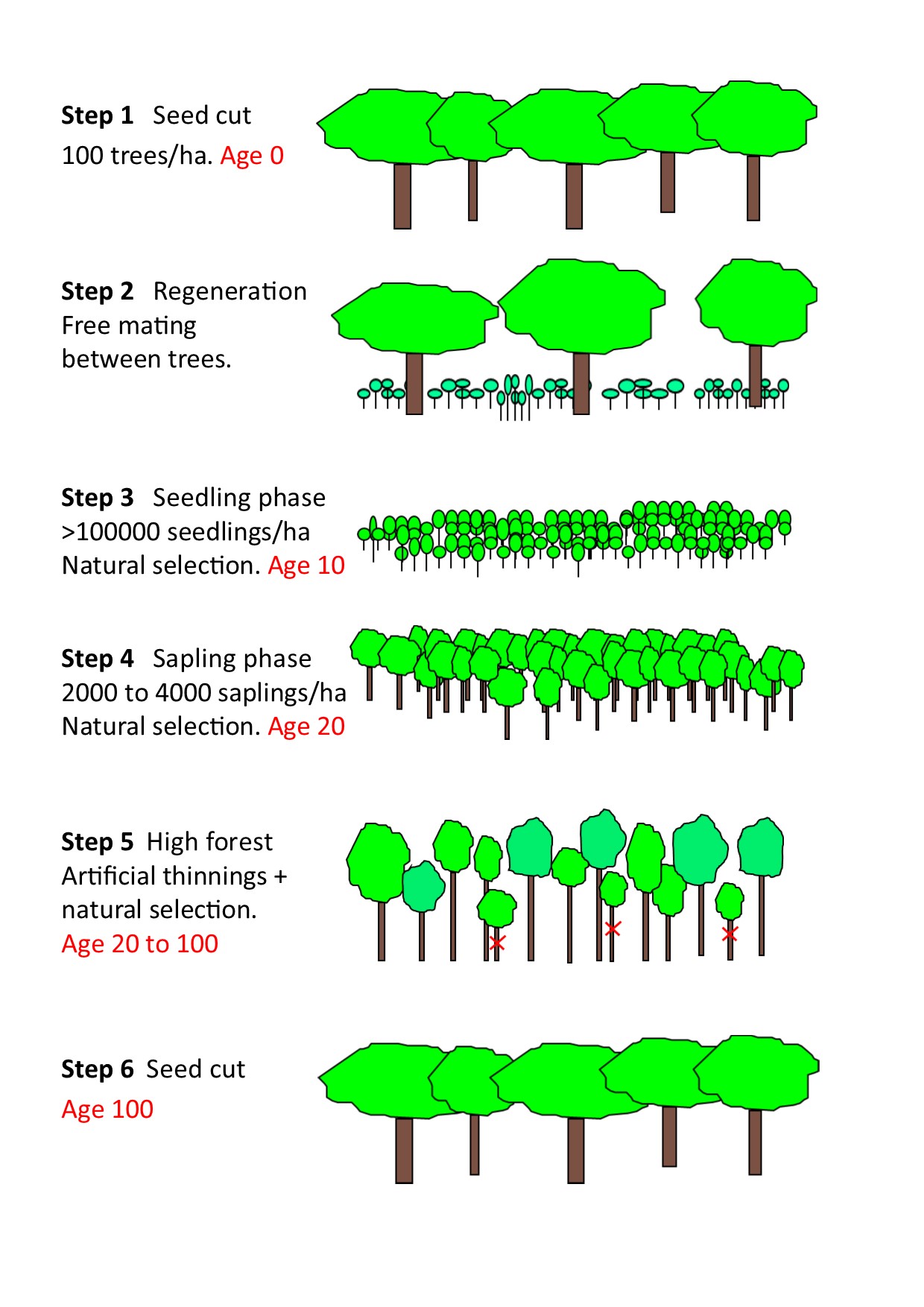


**Supplemental material S2.**

**Assessments of phenotypic traits**

1. Assessments in the G1 generation

1.1 Growth

At the final removal cut of G1 trees, at an age of about 100 years, we assessed numerous dimensional phenotypic traits. Cutting operations were performed in three lots in successive years (December 1998 to March 2001), to facilitate the recording of traits. Circumference (CIRC) at breast height was recorded before the trees were felled, and the total height (HGHT) of the trees was assessed after felling. We collected two 10 cm-wide sections (section 1 and 2) from the main stem, at a height of 1.30 m to 1.50 m from the ground, for subsequent laboratory assessments. Section 1 was used for the measurement of all annual ring widths along four radii in the four cardinal directions, and for assessing wood density (see the Structure - Wood density paragraph 1.5). Ring width (RWDTH) and ring surface area (RSURF) were recorded for each ring in each individual. Section 2 was then used for the extraction and analysis of secondary metabolites for the evaluation of wood composition (see the Defence: secondary metabolites paragraph 1.7).

1.2 Phenology

The dates at which male and female flowering (MFLW and FFLW) occurred on each single tree were determined by monitoring floral development every three days in the spring of 1990, every 14 days in the spring of 1991 and every 7 days in the spring of 1992 (Bacilieri *et al*., 1994). Floral development was recorded in the upper part of the crown, and classified into five classes, from early (1) to late (5) flowering stages, on the basis of observations made with a telescope at 25x or 40x magnification. From these observations, flowering dates for male and female flowers were calculated, separately, for 1990, 1991 and 1992 (date in Gregorian calendar days on which flowers reach stage 3, corresponding to the release of pollen from catkins for male flowers and receptive pistils, bright red in color, for female flowers). We monitored leaf unfolding (LU) on apical buds in April 2016, in the *ex situ* grafted conservation collection, by scoring the development stage of the buds (5 classes) according to a published protocol (Vitasse et al., 2009). LU was used to assess the phenological development of the vegetative apical bud. Two measurements were used: LUs, the score at each observation date, and LUd, the date (Gregorian calendar) on which unfolding occurred, derived from LUs. Leaf senescence (LS) was also assessed late in the 2016 season, in the *ex situ* collection. Between October 26 and November 21, the percentage of leaves turning yellow or falling was scored visually for each tree, and leaf senescence was considered complete when more than 50% of the crown turned yellow. LS is the date (in Gregorian calendar days) on which senescence was considered complete. We calculated growing season length (GSL) as the number of days from unfolding to senescense (GSL = LS - LUd). In January 1998 and February 2000, leaf retention (MAR for marcescence) within the tree canopy was assessed *in situ* by attributing a score from 0 to 5.

1.3 Physiology

Branches (about 50 cm long) were cut from the upper crown of the grafts located in the *ex situ* conservation plantation with a pole pruner, in the summer of 2016. Branches were wrapped in sealed plastic bags after collection, to prevent desiccation, and were transported to the laboratory. Leaf area was measured on six to eight leaves collected from these branches, with a desktop scanner (Expression 10000 XL, Epson, Japan) and WinFolia software (Regent Instruments Inc., Quebec, Canada), with averaging to obtained the mean leaf area (MLA). Leaves were then dried in an oven at 65 °C until a constant mass was reached. Specific leaf area (SLA) was then measured as the ratio of leaf area to dry mass. The same leaves were then used for the determination of carbon and nitrogen contents (C and N g/kg, respectively) and for the assessment of stable isotopic composition (^13^C and ^15^N, respectively), as described in a previous study (Torres-Ruiz et al., 2019), according to the formula:

$$\delta=\left( \frac{R_{sample}}{R_{standard}}-1 \right)*1000$$

Where δ is δ ^13^C or δ^15^N and *R* is the ^13^C/^12^C or ^15^N/^14^N ratio

1.4 Resilience

The resilience components of the trees were assessed by assessing the tree cambial growth response to a severe disturbance. This method involved comparisons of ring width before, during and after so-called “negative” pointer years in which the disturbance occurred (Lloret et al., 2011). We used the whole data set for the available tree rings for the 100-year period to identify seven negative pointer years for *Q. petraea* and 10 for *Q. robur* (see (Truffaut et al., 2017) for more details). We then calculated three resilience components: 1) resistance (RET): the inverse of ring width reduction during the disturbance; 2) recovery (REC): the increase in ring width after the disturbance relative to the minimum ring width during the disturbance, as an expression of the ability of tree growth to recover after a disturbance; and 3) resilience (REL): ring width after recovery relative to ring width before the disturbance, reflecting the ability of trees to return to pre-disturbance growth levels (Folke et al., 2004). Resilience components were derived from tree ring analysis for three periods in the lifetime of a tree: the juvenile period corresponding to the first 30 years of growth, the intermediate period corresponding to a tree age of 30 to 60 years, and the mature stage when trees were more than 60 years old.

1.5 Structure - Wood density

Two radial wood bars (6 cm thick) extending from the cambium to the core of the tree were randomly delineated and extracted from section 1 collected from the stem (see the Growth section). They were saturated by incubation in water for 48 hours and were then dried at 100 °C for 28 hours. The wood density (WD) of G1 trees was assessed as infradensity, by calculating the ratio of dry weight to the water-saturated volume of the bars (Guilley, 2000).

1.6 Leaf morphology

Leaf morphology data were extracted from a previous study (Kremer et al., 2002). The data collected concerned nine raw traits (LL: lamina length, PL: petiole length, LW: lobe width, SW: sinus width, WP: length of lamina at largest width, NL: number of lobes, NV: number of intercalary veins, BS: basal shape of the lamina, HR: pubescence) and five synthetic traits calculated from the nine raw traits (OB: lamina shape obversity, PR: petiole ratio, LDR: lobe depth ratio, PV: percentage venation, LWR: lobe width ratio). Data were available for five leaves collected from the upper part of the crown for each tree sampled *in situ*.

1.7 Defence: secondary metabolites

Wood metabolites were extracted from section 2 collected from the stem of the adult trees and analyzed. This involved the extraction of a 10 cm-wide diametral strip from the section, and the collection of a wood shaving from the two ends (excluding sapwood). Wood shavings from about 35 to 40 rings were used for subsequent extractions and analysis of the compounds present, by HPLC (for ellagitannins, Prida et al. (2006)) or GC/MS (for other volatile compounds, Prida et al. (2007)). In total, 21 compounds were identified. Ellagitannins are present in various parts of oak trees: heartwood, bark, leaves, seed (Moilanen, Koskinen, & Salminen, 2015; Mosedale, Charrier, Crouch, Janin, & Savill, 1996). *Q. robur* was found to be richer in ellagitannins than *Q. petraea* (Garcia-Estevez, Alcalde-Eon, Le Grottaglie, Rivas-Gonzalo, & Escribano-Bailon, 2015; Prida, Boulet, Ducousso, Nepveu, & Puech, 2006), whereas the opposite pattern was observed for some volatile compounds (whiskey lactone and eugenol) (Prida, Ducousso, Petit, Nepveu, & Puech, 2007). The contribution of these metabolites to wine maturation in oak barrels has been studied in detail (Cadahia, Varea, Munoz, de Simon, & Garcia-Vallejo, 2001; Michel et al., 2011; Viriot, Scalbert, Lapierre, & Moutounet, 1993). The ecological roles of these metabolites were little studied in the past, but recent developments have highlighted their possible involvement in causing oxidative stress in insect guts (Moilanen et al., 2016; Salminen & Karonen, 2011; Salminen, Karonen, & Sinkkonen, 2011). Ellagitannins and volatile compounds are thought to be involved in the resistance of trees to insects and pathogens, and in chemical signaling (Constabel, Yoshida, & Walker, 2014; Salminen, 2014; Tumlinson, 2014).

2. Assessments in the G2 generation

We also assessed a subset of 11 traits for the 370 *Q. petraea* and 390 *Q. robur* G2 trees for which sampling was performed *in situ*. In addition to the age-related differences between G1 (about 100 years old) and G2 (14 to 26 years old), there were also several differences in the procedures and protocols used to assess traits of G1 and G2 trees. For G2, total height (HGHT) was assessed *in situ* on standing living trees, using a vertex and circumference at breast height (CIRC) measured with a tape measure. Leaf unfolding (LUs) was monitored *in situ* on April 11-12 2017, by scoring the development stage of the apical bud (from 1 to 6). There were two few time points for the transformation of LUs into LUd . Wood density (WD) was measured *in situ* on the increment cores, by an X-ray image calibration procedure. Increment cores were exposed to X rays and then scanned with a microdensitometer, as described in a previous study (Polge & Nicholls, 1972). We used the mean of all cores for each individual, multiplied by 1000, to obtain a single measurement per individual. Physiology-related traits were measured after the collection of leaves *in situ* by the same protocol as for G1.

**Supplemental material S3.**

**Study populations, sampling and pedigree relationship between G2 and G1 trees.**

The study population consists of a mixed oak stand (*Q. petraea* and *Q. robur*) of natural origin located is the Petite Charnie State Forest (latitude: 48.086°N; longitude: 0.168°W) in North-West France. This stand is part of a long-term experiment aiming to monitor ecological and evolutionary processes in oak forests. The long-term study started with a seed cut (Step 1, Figure S1) in 1989, which left about 426 trees in place (about 90 to 100 years old).

**Figure S2**: Spatial distribution of G1 trees (scale is in meters)

The seed cut was followed by additional thinning operations (1992, 1993) and a final cut was practiced in between 1999 and 2001. The adult trees composing the stand from 1989 to 2001 correspond to our generation G1 (identified as cohort 1 in (Truffaut et al., 2017)). The final clear cut of the remaining 298 trees took place over a period of three years (1999, 2000 and 2001), to facilitate the harvesting and manipulation of log samples for later analyses of the wood and tree anatomy. Between 1989 and 2001, seeds originating from open pollination between the remaining adult trees germinated and developed into saplings. In total, about 11 000 saplings of *Q. robur* and 30 000 of *Q. petraea* (Truffaut *et al.* 2017, table 3) were assessed in 2014, when a demographic inventory was established. This sapling population constituted the G2 generation (cohort 2 in Truffaut *et al*., 2017), which was 14 to 28 years old when our investigation began in the summer of 2014. At that time, we sampled 2510 saplings from the G2 generation, corresponding to the systematic collection of one sapling every 3–6 m.

**Figure S3**: Spatial distribution of G2 saplings (scale is in meters)

A parentage analysis was conducted to determine parent-offspring relationships between G1 and G2 trees (Truffaut *et al*., 2017) based on 82 SNP loci, in CERVUS v.3.0.7 (Marshall, Slate, Kruuk, & Pemberton, 1998), with stringent parameters, assuming no errors in genotyping (a strict exclusion analysis: 0.0 error rate) and a high confidence level (95%). The parentage analysis showed that a very small number of adult trees did not contribute to the next generation (10 *Q. robur* and 3 *Q. petraea*). Based on the parentage analysis we identified a set of saplings for phenotypic assessment in G2, as a sample for estimating genetic variances/covariances. This set comprised offspring from all full-sib (FS) families detected in G2 (saplings for which the two parents were found within G1), and all half-sib families for which the identified parent was also the parent of at least one full-sib family. Overall, the G2 sample used for phenotypic assessments contained 370 *Q. petraea* and 390 *Quercus robur* saplings*.*

**Figure S4**: Spatial distribution of G2 saplings sampled for phenotypic assessment (scale is in meters)

**

Supplemental material S4.

**Spatially explicit mating model used to calculate effective reproductive success**

**Supplemental material S4.1. Description of the model**

The goal of this statistical analysis was to eliminate the effects of the spatial geometry of the experimental design from calculations of the reproductive success of all adult trees. The relative positions of the seedling sampling sites, adult trees and plot borders affect the observed reproductive success in CERVUS-like parentage analyses ((Oddou-Muratorio, Gauzere, Bontemps, Rey, & Klein, 2018)). As described by Oddou-Muratorio et al. (2018) and Tonnabel et al. (submitted), we used the MEMM_seedlings framework to infer, for each adult, an “effective fecundity”, referred to as “effective reproductive success” for the sake of simplicity in the manuscript. This inference was obtained from observations of sapling genotypes, assuming the locations of the sapling and the genotypes and locations of the adults to be known (Table S1).

The Bayesian approach used (first initiated in MEMM, (Klein, Desassis, & Oddou-Muratorio, 2008), and extended to MEMM_seedlings, (Oddou-Muratorio et al., 2018)) has the advantage of considering and estimating the total variances of male fecundity (*σ_M_²*) and female fecundity (*σ_F_²*) and of providing posterior estimates of male and female individual effective fecundities (*F_Mk_*, and *F_Fk_*, respectively) which appear as latent variables in the hierarchical model used (Figure S5). The MEMM methods also jointly estimate the dispersal and mating parameters required to eliminate spatial effects (e.g. for MEMM_seedlings: seed dispersal parameters *δ_s_, b_s_,* seed immigration rate *m_s_,* pollen dispersal parameters *δ_p_, b_p_,* selfing and pollen immigration rates *s* and *m_p_*). All estimates were obtained simultaneously with a Bayesian MCMC method implementing a Metropolis-Hastings algorithm (Table S2).

In this study, we needed to extend the previously published MEMM_seedling framework (Oddou-Muratorio et al., 2018) by adding the following components:

1. Two species with partial hybridization barriers to mating with each other, with different dispersal parameters, characterized by different allelic frequencies (Lagache, Klein, Ducousso, & Petit, 2014; Lagache, Klein, Guichoux, & Petit, 2013). The presence of a small number of hybrids in the adult population, and the expected subsequent immigration of seeds and pollen from hybrids outside the plots also led us to include parameters for the hybrid class.
2. Ghost trees (166 trees cut before 1998, see text, M&M section), i.e. adults at known positions within the plot but with unknown genotypes (trees that were cut during the removal cut in 1992, 1993). The genetic contributions of these ghost trees to the seedlings could not be distinguished from those of immigrant seeds and pollen. We therefore added a spatially explicit contribution of ghosts and spatially uniform numbers of immigrant seeds and immigrant pollen grains, to model spatially explicit immigration rates (e.g. according to Eq. 4 of Lagache et al., 2013 for pollen, duplicated here for immigrant seeds)
3. Neighborhood interactions (Burczyk, Adams, Birkes, & Chybicki, 2006). The large numbers of seedlings and adults resulted in execution times that were too long, largely due to the presence of ghost trees, which were “compatible” with all seedlings. We considered the spatially explicit contribution of ghost trees only in a restricted neighborhood of each seedling for seed contribution (50 m radius) and in a restricted neighborhood of each mother tree for the pollen contribution (150 m radius).

Finally the inferred male and female fecundities of each tree were averaged to provide a “reproductive success” variable that was analyzed with quantitative genetic methods in the manuscript. This analysis was limited to the 260 genotyped, to prevent potential artifacts due to the difference in inference quality (Figure S6, male fecundities).

**Table S1**. Data used to infer individual female and male fecundities by the MEMM_seedling approach, extended for this study

| Data | Known or observed? |  |
| --- | --- | --- |
| Locations of the 260 genotyped adult trees | Known | Exhaustively georeferenced in 1989 and surveyed in 1994 ((Kremer et al., 2002)) |
| Locations of the 166 ghost trees | Known | Exhaustively georeferenced in 1989 ((Kremer et al., 2002)) |
| Locations of the 2510 sampled seedlings | Known | Sampled on a regular grid in 2014 and georeferenced ((Truffaut et al., 2017)) |
| Species of the 260 genotyped adult trees | Known | Assigned from the SNPs ((Truffaut et al., 2017)) |
| Species of the 166 ghost trees | Known | Assigned from leaf morphology measurements ((Kremer et al., 2002)) |
| Genotypes of the 260 genotyped adult trees | Known | 82 SNPs read on material collected in 1994 ((Truffaut et al., 2017)) |
| Genotypes of the 2510 seedlings | Observed | 82 SNPs ((Truffaut et al., 2017)) |
| Allelic frequencies of *Quercus petraea* | Known | 82 SNPs ((Truffaut et al., 2017)) |
| Allelic frequencies of *Quercus robur* | Known | 82 SNPs ((Truffaut et al., 2017)) |

**Table S2**. Parameters included in the MEMM_seedling approach extended for this study. The estimates were obtained from the 10 MCMC runs and the confidence intervals were calculated from the posterior distribution of each parameter

| Parameter | Parameter name | Estimate | 95% confidence intervals |
| --- | --- | --- | --- |
| Seed dispersal |  |  |  |
| Mean dispersal distance QP | $\delta_{s,QP}$ | 8.42 m | (6.53 – 11.03) |
| Mean dispersal distance QR | $\delta_{s,QR}$ | 8.20 m | (6.08 – 8.75) |
| Mean dispersal distance HYB | $\delta_{s,HYB}$ | 3.95 m | (2.59 – 6.73) |
| Shape parameter QP | $b_{s,QP}$ | 0.27 | (0.23 – 0.34) |
| Shape parameter QR | $b_{s,QR}$ | 0.38 | (0.30 – 0.50) |
| Shape parameter HYB | $b_{s,HYB}$ | 0.37 | (0.27 – 0.52) |
| Seed migration🞯 |  |  |  |
| Number of immigrant seeds QP | $q_{s,QP}$ | 10^-4.5^ | (10^-5.1^ – 10^-3.7^) |
| Number of immigrant seeds QR | $q_{s,QR}$ | 10^-5.6^ | (10^-6.2^ – 10^-4.4^) |
| Number of immigrant seeds HYB | $q_{s,HYB}$ | 10^-5.8^ | (10^-6.7^ – 10^-5.3^) |
| Pollen dispersal* |  |  |  |
| Mean pollen dispersal distance | $\delta_{p}$ | 143.75 m | (110.08 – 206.87) |
| Pollen dispersal shape parameter | $b_{b}$ | 0.73 | (0.60 - 0.87) |
| Selfing and pollen migration rates |  |  |  |
| Amount of immigrant pollen QP | $q_{p,QP}$ | 10^1.7^ | (10^1.5^ – 10^1.8^) |
| Amount of immigrant pollen QR | $q_{p,QR}$ | 10^1.9^ | (10^1.7^ – 10^2.1^) |
| Amount of immigrant pollen HYB | $q_{p,HYB}$ | 10^2.3^ | (10^1.7^ – 10^2.1^) |
| Selfing rate | $s$ | 0.05 | (0.0002 – 0.029) |
| Hybridization barriers |  |  |  |
| QR on QP mothers | $h_{QP,QR}$ | 0.009 | (0.004 – 0.019) |
| QP on QR mothers | $h_{QR,QP}$ | 0.018 | (0.009 – 0.032) |
| Variances of fecundities |  |  |  |
| Var of female fecundity | $\sigma_{F}^{2}$ | 1.87 | (1.52 - 2.27) |
| Var of male fecundity | $\sigma_{M}^{2}$ | 1.40 | (0.96 – 2.09) |

🞯 The numbers of immigrant seeds and amounts of immigrant pollen are not informative *per se* because they are largely dependent on the numbers of seeds and amounts of pollen predicted from the inside and largely dependent on the tail of the dispersal kernel

* It would be possible to model different pollen dispersal kernels for the different species, but we chose to consider the same pollen dispersal kernel for both species, to increase robustness.

**Figure S5**. Structure of the hierarchical model used for Bayesian inference


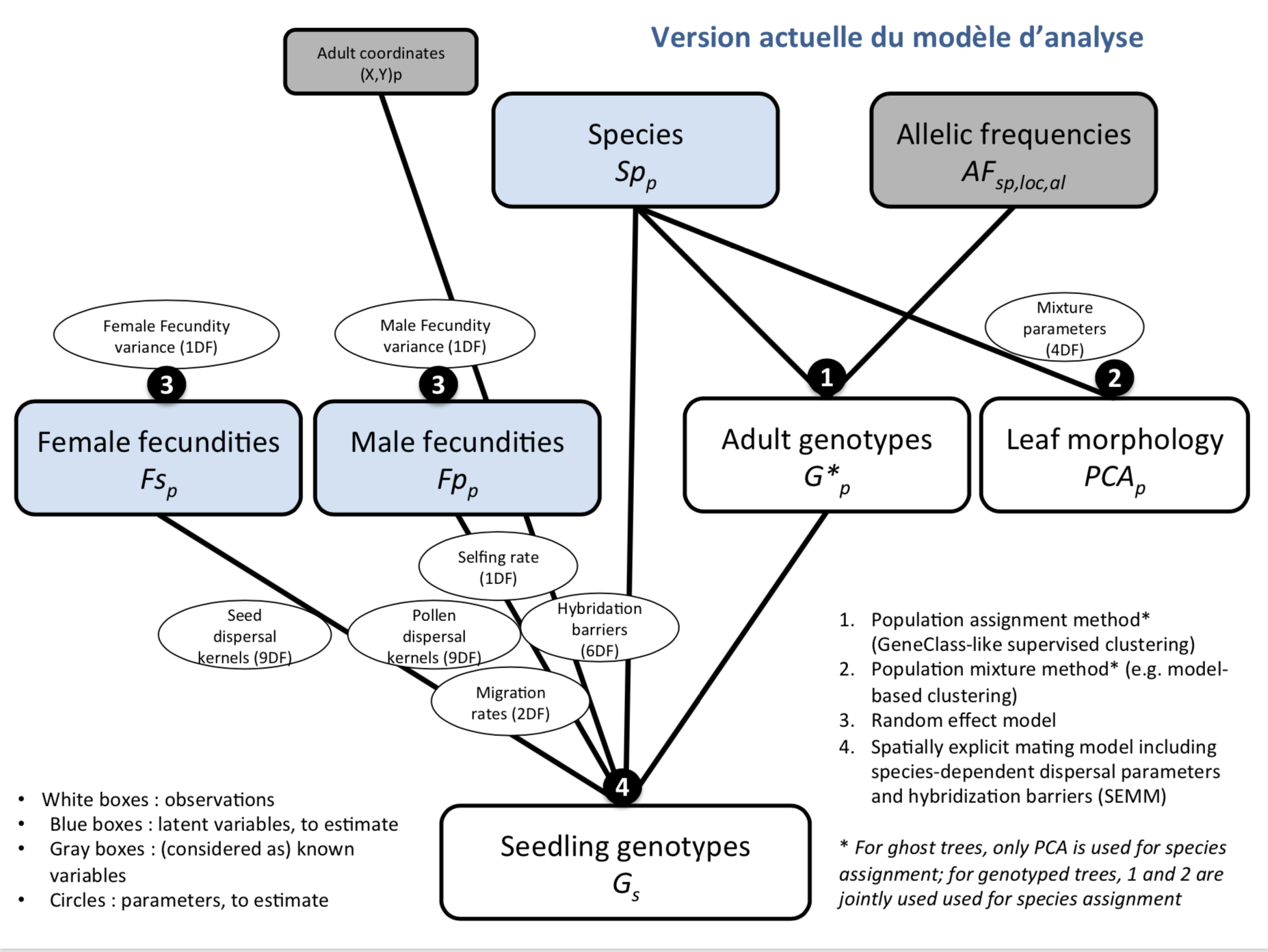


**Figure S6.** Inferred values for female (top) and male (bottom) individual fecundities. The 260 bars on the left correspond to genotyped trees and the 166 bars on the right correspond to ghost trees.

**Supplemental material S4.2.**

**Comparison of the effective reproductive success (estimated with MEMM) and realized reproductive success estimated by parentage analysis (CERVUS)**

We compared the effective reproductive success as estimated with MEMM and the realized reproductive success estimated from a categorical parentage analysis with CERVUS (Marshall et al., 1998). Top panels (Figure S7a and Figure S7b) represent realized reproductive success (y-axis) vs. effective reproductive success (x-axis) of *Q.petraea* and *Q. robur trees* with a color scale representing the residual value. Bottom panels (Figure S7c and Figure S7d) represent the spatial locations of trees and the trees with the largest residuals are labelled. These panels support what was expected. There is a clear correlation between both effective and realized reproductive success and outlier points correspond to cases where discrepancies are indeed expected due to peculiar spatial location of trees. Some particular trees exhibit a large discrepancy (potentially due to long distance dispersers, or very atypical locations in the plot) and trees showing the strongest under-evaluation of the reproductive success are located in the periphery of the plot (i.e. trees for which the reproductive success is less accurately estimated).

| **Figure S7a** Realized vs Effective reproductive success in *Q. petraea* | **Figure S7b** Realized vs Effective reproductive success in *Q. robur* |
| --- | --- |
| **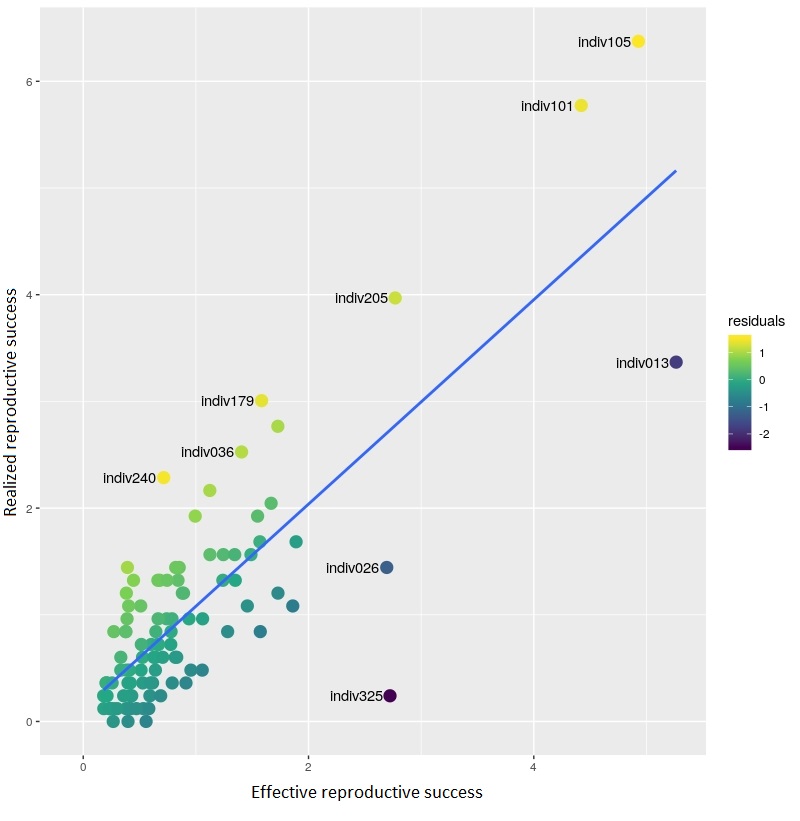** | **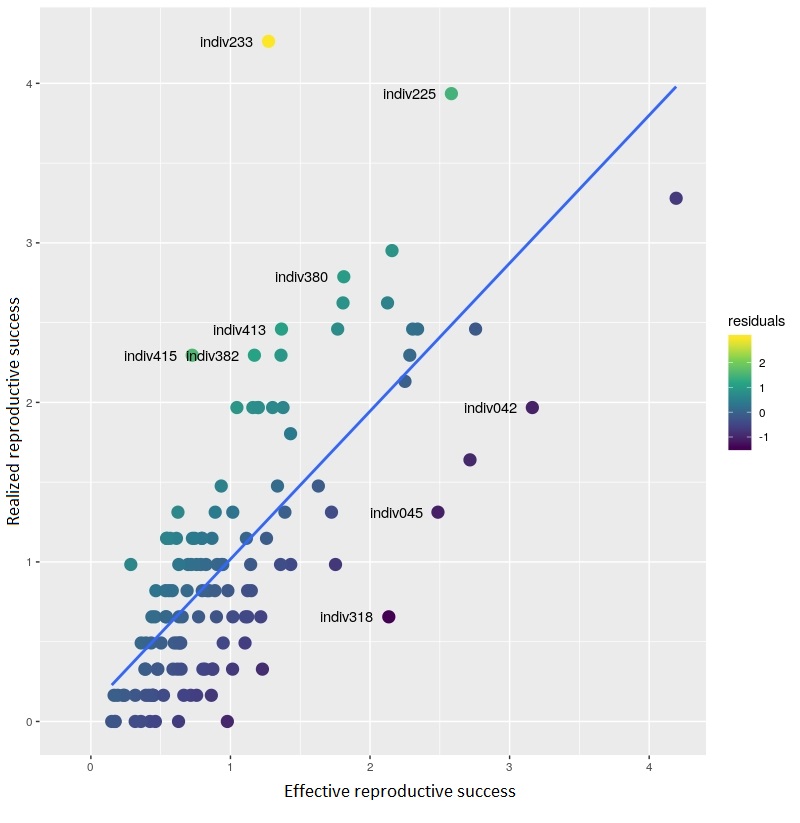** |
| **Figure S7c** Spatial position of G1 *Q. petraea* trees | **Figure S7d** Spatial position of G1 *Q. robur* trees |
| **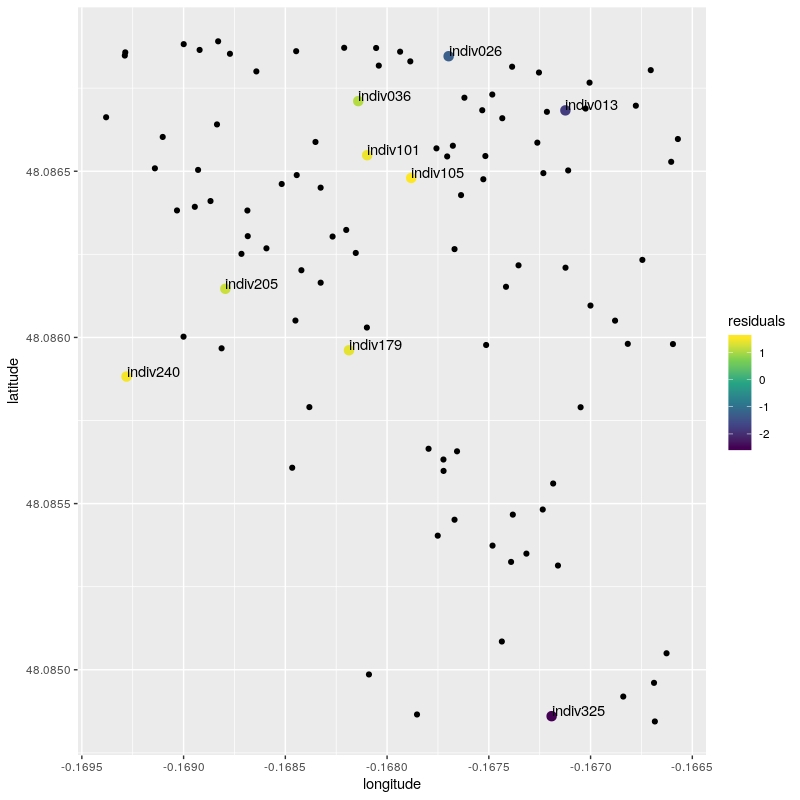** | **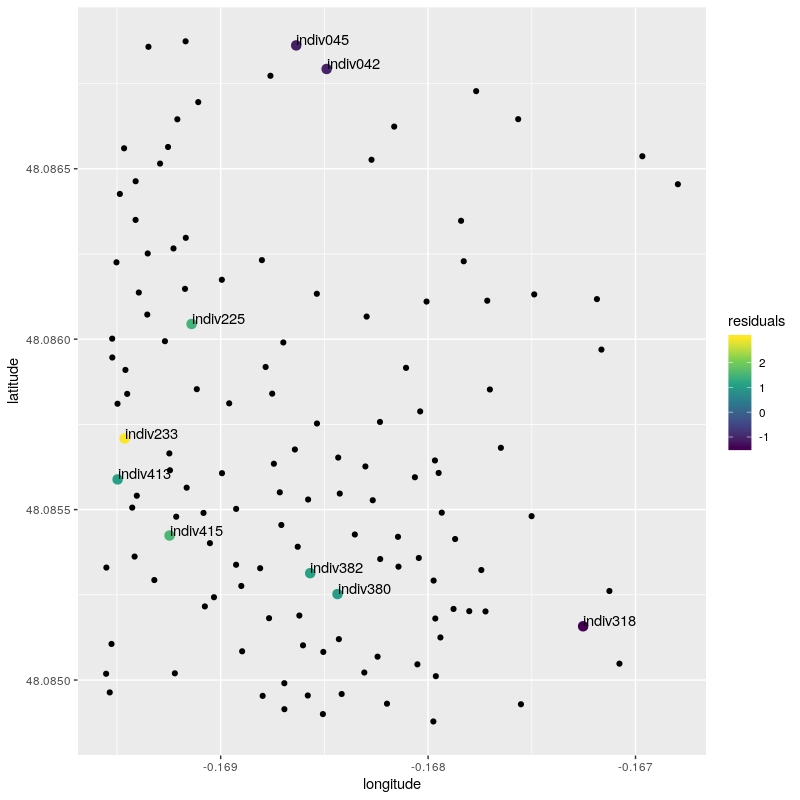** |

**Supplemental material S5**

**Variation of the annual water deficit during the study period (1989-2011(Vidal, Martin, Franchisteguy, Baillon, & Soubeyroux, 2010)).**

Soil water deficit was calculated with Biljou©, the daily water balance model for forests (Granier, Breda, Biron, & Villette, 1999)(https://appgeodb.nancy.inra.fr/biljou/en). This process-based model calculates daily water fluxes (interception, transpiration, evapotranspiration, drainage) and soil water content every day, from climatic data (precipitation, temperature, air humidity, wind speed and global radiation), soil parameters (maximum extractable water, bulk density, and water content at permanent wilting point) and stand parameters (leaf area index, fine root distribution). Climatic data were collected from the SAFRAN reanalysis for 1959 to 2011 (Vidal et al., 2010), the available soil water content was set at 100 mm and the LAI was set at 6 m²/m². From 1989 onwards, the mean soil water stress index (incorporating both the duration and intensity of soil water deficit) increased by 21% relative to the levels observed between 1959 and 1988. The droughts in 1989, 1990, 1996 and 2005 were exceptional for the whole period.

**Figure S7**: Variation of annual water deficit during the study period


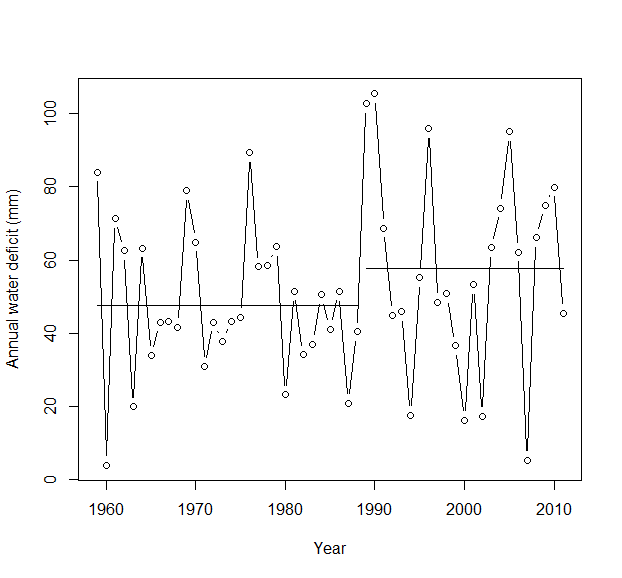


**Supplemental material S6**

**Quadratic phenotypic selection gradients (γ) and p value corresponding to the statistical test (*p*val(γ))**

|  |  | *Quercus petraea* | | *Quercus robur* | |
| --- | --- | --- | --- | --- | --- |
|  |  | γ | *p*val(γ) | γ | *p*val(γ) |
| Growth | CIRC | 0.155 | 0.388 | -0.054 | 0.943 |
|  | HGHT | 0.014 | 0.527 | -0.011 | 0.752 |
|  | RSURF | 0.038 | 0.901 | -0.108 | 0.375 |
|  | RWDTH | 0.156 | 0.643 | -0.060 | 0.802 |
| Phenology | LU | -0.192 | 0.100 | -0.036 | 0.450 |
|  | LS | -0.020 | 0.712 | -0.005 | 0.536 |
|  | GSL_ | -0.013 | 0.863 | -0.132 | 0.098 |
|  | FFLW | 0.044 | 0.721 | 0.112 | 0.294 |
|  | MFLW | -0.681 | 0.413 | 0.043 | 0.830 |
|  | MAR | 0.317 | 0.152 | 0.110 | 0.372 |
| Physiology | C | 0.051 | 0.855 | 0.114 | 0.179 |
|  | C/N | 0.012 | 0.662 | 0.118 | 0.017 |
|  | δ^15^C | -0.154 | 0.114 | -0.102 | 0.650 |
|  | δ^15^N | -0.172 | 0.520 | -0.190 | 0.269 |
|  | MLA | 0.102 | 0.353 | -0.068 | 0.529 |
|  | N | 0.228 | 0.286 | 0.125 | 0.054 |
|  | SLA | -0.199 | 0.234 | -0.029 | 0.848 |
| Resilience | REC | -0.133 | 0.469 | -0.015 | 0.541 |
|  | REL | -0.015 | 0.800 | 0.018 | 0.751 |
|  | RET | -0.034 | 0.752 | 0.025 | 0.699 |
| Structure | WD | 0.064 | 0.683 | -0.104 | 0.148 |
| Leaf morphology | BS | -0.073 | 0.235 | 0.078 | 0.542 |
|  | HR | 0.014 | 0.526 | -0.054 | 0.417 |
|  | LDR | 0.006 | 0.966 | 0.059 | 0.899 |
|  | LL | -0.129 | 0.180 | 0.043 | 0.395 |
|  | LW | -0.166 | 0.313 | 0.054 | 0.172 |
|  | LWR | -0.028 | 0.939 | -0.007 | 0.951 |
|  | NL | 0.019 | 0.432 | 0.002 | 0.850 |
|  | NV | 0.014 | 0.288 | -0.023 | 0.244 |
|  | OB | 0.079 | 0.708 | 0.016 | 0.544 |
|  | PR | -0.158 | 0.167 | 0.059 | 0.395 |
|  | PV | 0.023 | 0.439 | -0.038 | 0.266 |
|  | SW | -0.357 | 0.063 | 0.052 | 0.438 |
|  | WP | -0.155 | 0.407 | 0.033 | 0.453 |
| Defence | CNFL | 0.094 | 0.411 | -0.031 | 0.585 |
|  | CSTG | -0.112 | 0.708 | 0.024 | 0.717 |
|  | CSTL | -0.209 | 0.144 | 0.063 | 0.387 |
|  | CWSK | 0.187 | 0.567 | -0.019 | 0.488 |
|  | EGNL | 0.129 | 0.193 | 0.013 | 0.809 |
|  | ELAC | -0.057 | 0.387 | -0.072 | 0.222 |
|  | ELTOT | -0.111 | 0.447 | 0.059 | 0.803 |
|  | GRDN | 0.080 | 0.902 | 0.030 | 0.849 |
|  | MVL | -0.094 | 0.463 | -0.112 | 0.295 |
|  | PNTL | -0.153 | 0.144 | 0.009 | 0.813 |
|  | ROBA | 0.023 | 0.777 | -0.056 | 0.371 |
|  | ROBB | 0.213 | 0.047 | 0.009 | 0.809 |
|  | ROBC | 0.084 | 0.586 | -0.051 | 0.603 |
|  | ROBD | 0.170 | 0.219 | 0.037 | 0.424 |
|  | ROBE | -0.019 | 0.871 | 0.056 | 0.274 |
|  | SYRG | 0.034 | 0.582 | -0.079 | 0.071 |
|  | TWSK | 0.044 | 0.575 | 0.035 | 0.809 |
|  | VNL | 0.062 | 0.592 | -0.212 | 0.010 |
|  | VSCG | -0.069 | 0.675 | -0.047 | 0.769 |
|  | VSCL | -0.112 | 0.296 | 0.072 | 0.275 |
|  | X2PHL | -0.041 | 0.486 | -0.003 | 0.638 |

**Supplemental material S7.**

**Exposure of bud burst to selection driven by occurrences of late frost.**

We checked the occurrence of late damaging frosts during the recruitment (+seedling and sapling) period of the G2 trees (1989-2014) by mining the temperature records provided by the SAFRAN data base of Meteo France. SAFRAN provides interpolated data of temperatures at a 8kmx8km grid across the country, based on data generated by climatic recording stations installed by Meteo France. We extracted the daily minimum temperature data of the mesh containing the Petite Charnie stand. According to Liepe et al. (1993), frost damages on flushing oak seedlings occur when temperatures drop below -3°C. Furthermore as observed in our phenological survey during spring 1991, 1992 and 1993, bud flushing locally occurs later than April 1st. We thus retrospectively identified possible occurrences of late frost by pointing years when minimum temperatures dropped below -2°C after April 1st. Such events were rather frequent before 1990, but occurred only twice during the period of interest, in 1996 and 2003 and at a moderate freezing temperature (-2°C and -3°C). We conclude from this survey that seedlings of G2 were not exposed to severe frost damages during their juvenile phase, up to 2014.

**Figure S8** Occurrences of damaging frosts (temperature below -2°C) after April 1st in La Petite Charnie stand. DOY indicates the Julian day of the year when the frost occurred.


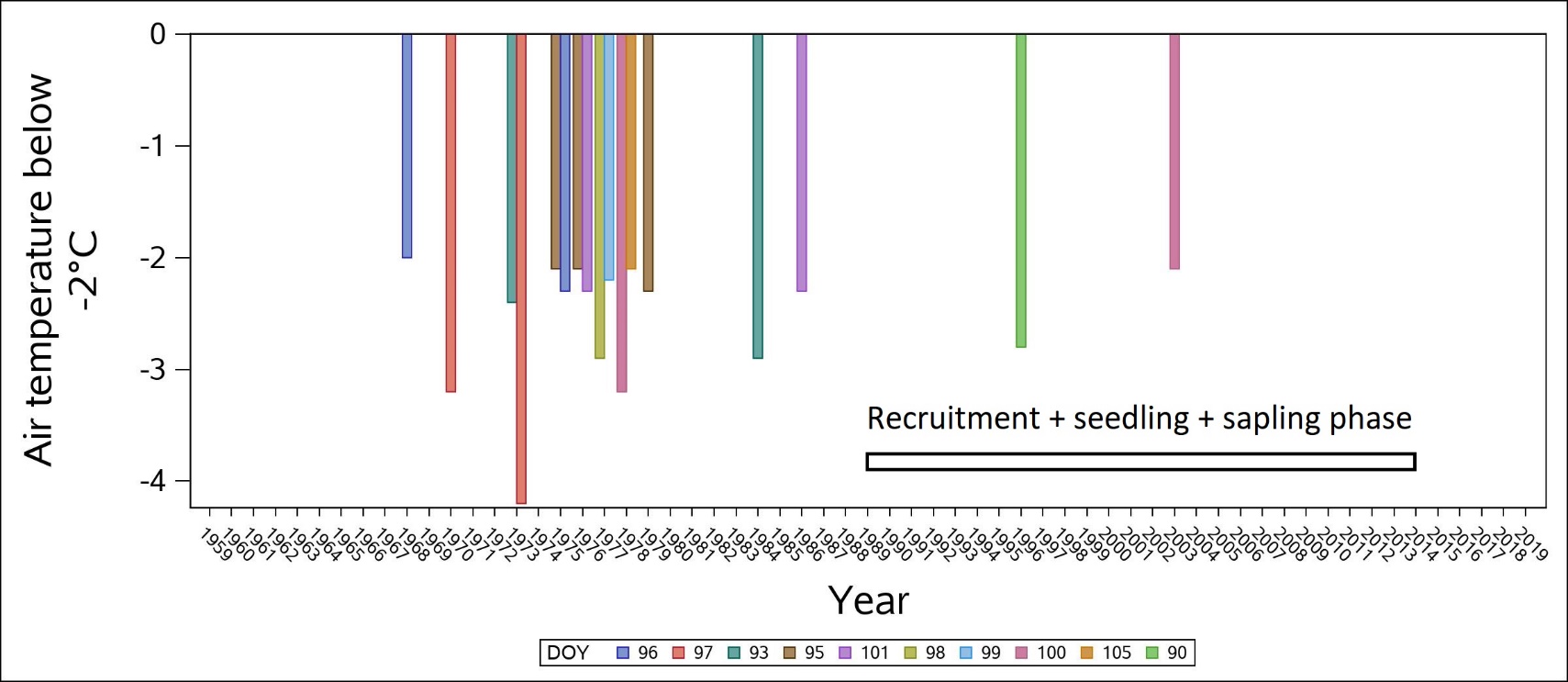


**Literature cited**

Bacilieri, R., Ducousso, A., Petit, R. J., & Kremer, A. (1996). Mating system and asymmetric hybridization in a mixed stand of European oaks. *Evolution, 50*(2), 900-908. doi:10.2307/2410861

Burczyk, J., Adams, W. T., Birkes, D. S., & Chybicki, I. J. (2006). Using genetic markers to directly estimate gene flow and reproductive success parameters in plants on the basis of naturally regenerated seedlings. *Genetics, 173*(1), 363-372. doi:10.1534/genetics.105.046805

Cadahia, E., Varea, S., Munoz, L., de Simon, B. F., & Garcia-Vallejo, M. C. (2001). Evolution of ellagitannins in Spanish, French, and American oak woods during natural seasoning and toasting. *Journal of Agricultural and Food Chemistry, 49*(8), 3677-3684. doi:10.1021/jf010288r

Constabel, C. P., Yoshida, K., & Walker, V. (2014). *Diverse Ecological Roles of Plant Tannins: Plant Defense and Beyond* (Vol. 4).

Folke, C., Carpenter, S., Walker, B., Scheffer, M., Elmqvist, T., Gunderson, L., & Holling, C. S. (2004). Regime shifts, resilience, and biodiversity in ecosystem management. *Annual Review of Ecology Evolution and Systematics, 35*, 557-581. doi:10.1146/annurev.ecolsys.35.021103.105711

Garcia-Estevez, I., Alcalde-Eon, C., Le Grottaglie, L., Rivas-Gonzalo, J. C., & Escribano-Bailon, M. T. (2015). Understanding the ellagitannin extraction process from oak wood. *Tetrahedron, 71*(20), 3089-3094. doi:10.1016/j.tet.2014.10.047

Granier, A., Breda, N., Biron, P., & Villette, S. (1999). A lumped water balance model to evaluate duration and intensity of drought constraints in forest stands. *Ecological Modelling, 116*(2-3), 269-283. doi:10.1016/s0304-3800(98)00205-1

Guilley, E. (2000). *La densité du bois de chêne sessile (Quercus petraea Liebl.). .* Ecole Nationale du Génie Rural des Eaux et des Forêts, Nancy.

Jarret, P. (2004). *Chênaie atlantique*: Lavoisier.

Klein, E. K., Desassis, N., & Oddou-Muratorio, S. (2008). Pollen flow in the wildservice tree, *Sorbus torminalis* (L.) Crantz. IV. Whole interindividual variance of male fecundity estimated jointly with the dispersal kernel. *Molecular Ecology, 17*(14), 3323-3336. doi:10.1111/j.1365-294X.2008.03809.x

Kremer, A., Dupouey, J. L., Deans, J. D., Cottrell, J., Csaikl, U., Finkeldey, R., . . . Badeau, V. (2002). Leaf morphological differentiation between *Quercus robur* and *Quercus petraea* is stable across western European mixed oak stands. *Annals of Forest Science, 59*(7), 777-787. doi:10.1051/forest:2002065

Lagache, L., Klein, E. K., Ducousso, A., & Petit, R. J. (2014). Distinct male reproductive strategies in two closely related oak species. *Molecular Ecology, 23*(17), 4331-4343. doi:10.1111/mec.12766

Lagache, L., Klein, E. K., Guichoux, E., & Petit, R. J. (2013). Fine-scale environmental control of hybridization in oaks. *Molecular Ecology, 22*(2), 423-436. doi:10.1111/mec.12121

Liepe, K. (1993). Growth-chamber trial on frost hardiness and field trial on flushing of sessile oak (*Quercus petraea* Liebl.). *Annales des Sciences Forestières,* 50 (Suppl. 1), 208s-214s.

Marshall, T. C., Slate, J., Kruuk, L. E. B., & Pemberton, J. M. (1998). Statistical confidence for likelihood-based paternity inference in natural populations. *Molecular Ecology, 7*(5), 639-655. doi:10.1046/j.1365-294x.1998.00374.x

Michel, J., Jourdes, M., Silva, M. A., Giordanengo, T., Mourey, N., & Teissedre, P. L. (2011). Impact of concentration of ellagitannins in oak wood on their levels and organoleptic influence in red wine. *Journal of Agricultural and Food Chemistry, 59*(10), 5677-5683. doi:10.1021/jf200275w

Moilanen, J., Karonen, M., Tahtinen, P., Jacquet, R., Quideau, S., & Salminen, J. P. (2016). Biological activity of ellagitannins: Effects as anti-oxidants, pro-oxidants and metal chelators. *Phytochemistry, 125*, 65-72. doi:10.1016/j.phytochem.2016.02.008

Moilanen, J., Koskinen, P., & Salminen, J. P. (2015). Distribution and content of ellagitannins in Finnish plant species. *Phytochemistry, 116*, 188-197. doi:10.1016/j.phytochem.2015.03.002

Mosedale, J. R., Charrier, B., Crouch, N., Janin, G., & Savill, P. S. (1996). Variation in the composition and content of ellagitannins in the heartwood of European oaks (*Quercus robur* and *Q. petraea*). A comparison of two French forests and variation with heartwood age. *Annales Des Sciences Forestieres, 53*(5), 1005-1018. doi:10.1051/forest:19960508

Oddou-Muratorio, S., Gauzere, J., Bontemps, A., Rey, J. F., & Klein, E. K. (2018). Tree, sex and size: Ecological determinants of male vs. female fecundity in three *Fagus sylvatica* stands. *Molecular Ecology, 27*(15), 3131-3145. doi:10.1111/mec.14770

Polge, H., & Nicholls, J. (1972). Quantitative radiography and the densitometric analysis of wood. *Wood Science, 5*, 51-59.

Prida, A., Boulet, J. C., Ducousso, A., Nepveu, G., & Puech, J. L. (2006). Effect of species and ecological conditions on ellagitannin content in oak wood from an even-aged and mixed stand of *Quercus robur* L. and *Quercus petraea* Liebl. *Annals of Forest Science, 63*(4), 415-424. doi:10.1051/forest:2006021

Prida, A., Ducousso, A., Petit, R. J., Nepveu, G., & Puech, J. L. (2007). Variation in wood volatile compounds in a mixed oak stand: strong species and spatial differentiation in whisky-lactone content. *Annals of Forest Science, 64*(3), 313-320. doi:10.1051/forest:2007008

Salminen, J. P. (2014). *The Chemistry and Chemical Ecology of Ellagitannins in Plant-Insect Interactions: From Underestimated Molecules to Bioactive Plant Constituents* (Vol. 4).

Salminen, J. P., & Karonen, M. (2011). Chemical ecology of tannins and other phenolics: we need a change in approach. *Functional Ecology, 25*(2), 325-338. doi:10.1111/j.1365-2435.2010.01826.x

Salminen, J. P., Karonen, M., & Sinkkonen, J. (2011). Chemical ecology of tannins: recent developments in tannin chemistry reveal new structures and structure-activity patterns. *Chemistry-a European Journal, 17*(10), 2806-2816. doi:10.1002/chem.201002662

Torres-Ruiz, J. M., Kremer, A., Carins-Murphy, M. R., Brodribb, T. J., Lamarque, L. J., Truffaut, L., . . . Delzon, S. (2019). Genetic differentiation in functional traits among European sessile oak populations growing in a common garden. *Journal of Ecology (submitted)*. Mise à jour nécessaire: soit 2019 et il faut donner la réf, soit toujours pas publié = 2020

Truffaut, L., Chancerel, E., Ducousso, A., Dupouey, J. L., Badeau, V., Ehrenmann, F., & Kremer, A. (2017). Fine-scale species distribution changes in a mixed oak stand over two successive generations. *New Phytologist, 215*(1), 126-139. doi:10.1111/nph.14561

Tumlinson, J. H. (2014). The importance of volatile organic compounds in ecosystem functioning. *Journal of Chemical Ecology, 40*, 212-213.

Vidal, J. P., Martin, E., Franchisteguy, L., Baillon, M., & Soubeyroux, J. M. (2010). A 50-year high-resolution atmospheric reanalysis over France with the Safran system. *International Journal of Climatology, 30*(11), 1627-1644. doi:10.1002/joc.2003

Viriot, C., Scalbert, A., Lapierre, C., & Moutounet, M. (1993). Ellagitannins and lignins in aging of spirits in oak barrels. *Journal of Agricultural and Food Chemistry, 41*(11), 1872-1879. doi:10.1021/jf00035a013

Vitasse, Y., Delzon, S., Dufrene, E., Pontailler, J. Y., Louvet, J. M., Kremer, A., & Michalet, R. (2009). Leaf phenology sensitivity to temperature in European trees: Do within-species populations exhibit similar responses? *Agricultural and Forest Meteorology, 149*(5), 735-744. doi:10.1016/j.agrformet.2008.10.019
